# Intermediary conformations linked to the directionality of the aminoacylation pathway of nonribosomal peptide synthetases†

**DOI:** 10.1039/d0cb00220h

**Published:** 2021-03-04

**Authors:** Florian Mayerthaler, Anna-Lena Feldberg, Jonas Alfermann, Xun Sun, Wieland Steinchen, Haw Yang, Henning D. Mootz

**Affiliations:** Institute of Biochemistry, Department of Chemistry and Pharmacy, University of Muenster Münster Germany Henning.Mootz@uni-muenster.de; Department of Chemistry, Princeton University Princeton New Jersey USA; SYNMIKRO Research Center & Faculty of Chemistry, Philipps-University Marburg Germany

## Abstract

Nonribosomal peptide synthetases (NRPSs) are multifunctional megaenzymes that govern the stepwise biosynthesis of pharmaceutically important peptides. In an ATP-dependent assembly-line mechanism dedicated domains are responsible for each catalytic step. Crystal structures have provided insight into several conformations of interacting domains. However, the complete picture in solution of how domain dynamics and the timing of conformational changes effect a directional biosynthesis remains only poorly understood and will be important for the efficient reprogramming of NRPSs. Here we dissect the multiple conformational changes associated with the adenylation and thiolation reactions of the aminoacylation pathway under catalytic conditions. We used pyrophosphate (PP_*i*_) to biochemically drive the conformational changes backward and forward while performing an online monitoring with a Förster resonance energy transfer (FRET) didomain sensor, consisting of adenylation (A) and peptidyl-carrier protein (PCP) domains. Notably, we found aminoacyl thioester formation to efficiently occur in the presence of PP_*i*_ even at millimolar concentrations, despite the chemically and conformationally reversing effect of this metabolite and by-product. This finding settles conflicting reports and explains why intracellular PP_*i*_ concentrations do not impair NRP biosynthesis. A conserved amino acid was identified to be important for the mechanism under these conditions. FRET time-course analyses revealed that the directionality of the aminoacylation catalysis is correlated with conformational kinetics. Complemented by equilibrium hydrogen–deuterium exchange (HDX) analyses, our data uncovered the existence of at least one new intermediary conformation that is associated with the rate-determining step. We propose an expanded model of conformational changes in the NRPS aminoacylation pathway.

## Abstract 2

In-solution analysis of conformational changes of NRPS adenylation and peptidyl-carrier protein domains under catalytic conditions reveals a new intermediary conformation.

## Graphical abstract

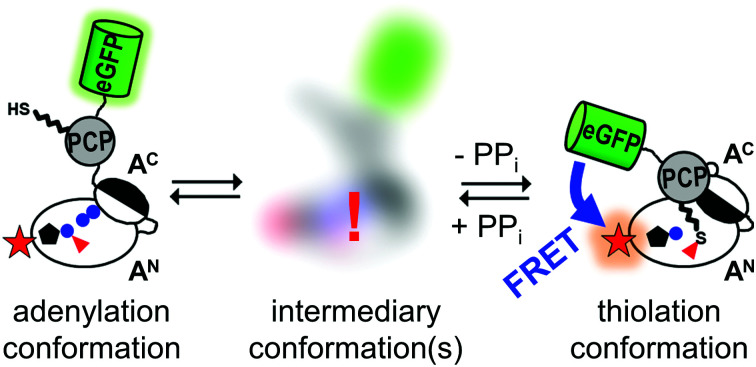

## Introduction

Nonribosomal peptide synthetases (NRPSs) are large multifunctional enzymes that are responsible for the assembly of a plethora of chemically diverse peptide secondary metabolites in bacterial and fungal microorganisms.^1–3^ Many of these nonribosomal peptides (NRPs) show strong biological activity and serve as important pharmaceuticals, such as the antibiotic vancomycin, the immune-suppressant cyclosporine and the antitumor drug bleomycin. Given the antibiotic resistance crisis, a detailed understanding of their underlying biosynthetic mechanisms becomes increasingly important to help in facilitating the exploitation of NRPS templates by combinatorial approaches for the discovery of new compounds.^1–5^

The structural and functional organization of NRPSs is reminiscent of an assembly line. Arrays of single domains act together in a sequential manner to synthesize the peptide product from N to C terminus under the consumption of ATP. In a typical linear NRPS,^6^ all domains required for the incorporation of one amino acid are arranged in a module. The number and order of modules determines the sequence of the final product.^2,7–9^ In a two-step aminoacylation pathway, the adenylation (A) domain first selects and activates the amino acid to the aminoacyl adenylate. In the second reaction the aminoacyl moiety is transferred onto the terminal thiol group of the 4′-phosphopantetheine prosthetic group (Ppant) of the peptidyl-carrier protein (PCP) domain. The PCP domain translocates the covalently bound aminoacyl or peptidyl thioesters as biosynthetic intermediates to other catalytic domains. The condensation (C) domain catalyzes peptide bond formation between an aminoacyl moiety and an upstream aminoacyl or peptidyl group bound on the PCPs of two adjacent modules. Optional domains such as epimerization (E) or *N*-methylation (M) domains lead to a modified amino acid at the respective position. The final off-loading of the peptidyl chain from the NRPS template is facilitated by thioesterase (TE) or C domains, typically located at the last module, and can result in cyclic, branched cyclic or linear peptide backbones.^1–3^

**Fig. 1:**
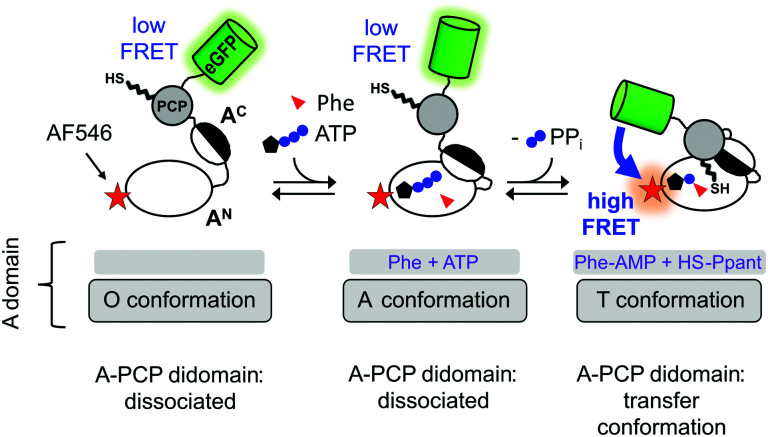
Current model of domain alternation and design of the A-PCP sensor. The A-PCP didomain of GrsA, the first module of the gramicidin S NRPS, is fused to the C terminus of the PCP with eGFP as the donor fluorophore, which replaces the native E domain in the full-length GrsA module. The acceptor fluorophore AlexaFluor546 (AF546) is conjugated as a maleimide to a single cysteine (N152C) in the A^N^ domain (Fig. S1, ESI†).

A central intriguing and still insufficiently answered question is how the multidomain catalysis of an NRPS template is coordinated to achieve the stepwise and directional peptide assembly. Significant conformational changes have been suggested from crystal structures that help explain how the PCP domain translocates across the large distances between the catalytic domains to allow binding of Ppant in the respective active sites.^10–14^ Furthermore, the A domain itself undergoes conformational changes that are coupled to ATP binding and consumption, as illustrated in Fig. 1 in the context of our A-PCP Förster resonance energy transfer (FRET) sensor described below. In the absence of substrates or ligands, the large N-terminal (A^N^) and small C-terminal (A^C^) subdomains are in an open conformation (O conformation). Upon binding of ATP/Mg^2+^ and the amino acid the A^C^ closes down on the A^N^ domain to give the adenylation conformation (A conformation) with the conserved A10 motif^2^ of the A^C^ domain in the active site. This conformation is adopted for catalysis of aminoacyl-AMP and PP_*i*_ formation. According to the domain alternation model^15^ the A^C^ then rotates by 140° relative to the A^N^ to adopt the closed thiolation conformation (T conformation) of the A domain,^15–17^ in which the conserved A8 motif^2^ of the A^C^ subdomain enters the active site and extrudes PP_*i*_. In the T conformation, the A domain is competent for binding the PCP domain, because the A^N^ and A^C^ subdomains form a composite interface for PCP binding and a narrow tunnel through which the Ppant group can reach into the active site for the aminoacyl transfer.^18–20^ We refer to the productive state of the A-PCP didomain with PCP bound to the A domain as the transfer conformation^21^ (Fig. 1). Thus, the A domain adopts the same overall structure in both the T and transfer conformations, but the former describes only the A domain, whereas the latter refers to the A-PCP didomain ensemble. It is not known if PCP binding to the A domain occurs before or after adoption of the T conformation.

While crystal structures of whole modules have been instrumental for our current understanding of conformations adopted along the reaction coordinate of NRP assembly,^12,18,22,23^ these static snapshots cannot unravel the dynamics of the conformational changes. Crystal structures may also miss conformations that are less favorable to crystallization and may instead highlight artificially stabilized conformations due to crystal packing effects.

To study NRPS conformational changes in real time in solution and under catalytic conditions, we have previously reported an A-PCP didomain FRET sensor (Fig. 1).^21^ From the responses of the A-PCP sensor to the addition of substrates or ligands in bulk measurements of protein populations unique insights could be gained into conformational changes and dynamics accompanying the chemical states of the enzyme.^21^ Under certain experimental conditions the enzyme population was shown to represent a mixture of at least two conformational states, suggesting the possibility of dynamic equilibria. The conserved lysine residues in the A10 and A8 motifs^2^ of the A domain are important for the ordered progression from the open to the two closed A and T conformations, respectively. Aminoacylation of the Ppant group effects the binding of the PCP to the A domain in a post-transfer conformation, effectively representing a product-inhibited state. This transfer conformation can be reversed by adding PP_*i*_ at high concentrations (∼1–10 mM), because PP_*i*_ binding competes with the interaction between the A^N^ and A^C^ subdomains by pushing the A8 motif out of the active site. These results underlined that studying NRPSs with a suitable FRET sensor is very promising to unravel conformational changes in solution and address the open questions in this regard.

Notably, the conformational change induced by excess PP_*i*_ reiterated the unanswered question of how the aminoacylation pathway can be upheld in the producing cell. Given that the intracellular PP_*i*_ concentrations are estimated to be between 0.5 and 6.0 mM in various bacteria,^24–26^ the reversing effect of PP_*i*_ on both the conformational equilibrium and the chemical adenylation reaction should affect the NRPS-mediated biosynthesis. Indeed, previous reports suggested a complete or strong impairment when PP_*i*_ was added to gramicidin S synthetase I, or GrsA,^27,28^ or to entire bacterial cells,^29^ although the molecular details were not studied.

Here, we have studied the functional and conformational effects of PP_*i*_ on the aminoacylation pathway to address these open questions. We show that aminoacylation as well as peptide elongation in an NRPS model system can occur with surprising efficiency in the presence of PP_*i*_ at high concentrations, and identify an important residue for this mechanism. Furthermore, a detailed kinetic analysis of the A-PCP conformational dynamics in solution using the A-PCP FRET sensor as well as hydrogen–deuterium exchange data call for at least one new intermediary (I) conformation in a revised domain alternation model. Finally, we propose a possible functional role for a previously reported orphan crystal structure with so far unrecognized relevance as an I conformation required in PCP binding to the A domain to adopt the transfer conformation.

## Results

### Kinetics of conformational changes observed following the addition and removal of PP_*i*_ to the A-PCP sensor point to an unknown slow step in the productive A-PCP pathway

We aimed to explore the same previously reported and validated A-PCP sensor for obtaining a more detailed picture of the catalytic and conformational interplay of the A and PCP domains in the two-step aminoacylation pathway. Specifically, we were interested to understand the effect of PP_*i*_ at high concentration on reversing conformational and chemical equilibria. Derived from the prototypic GrsA, the first module of the gramicidin S NRPS, the A-PCP sensor was extensively characterized in our previous study^21^ to specifically report with an increased FRET ratio on the adoption of the transfer conformation. In this state the donor and acceptor fluorophores, enhanced green fluorescent protein (eGFP) and AlexaFluor546 (AF546), come in close proximity (Fig. 1).

**Fig. 2:**
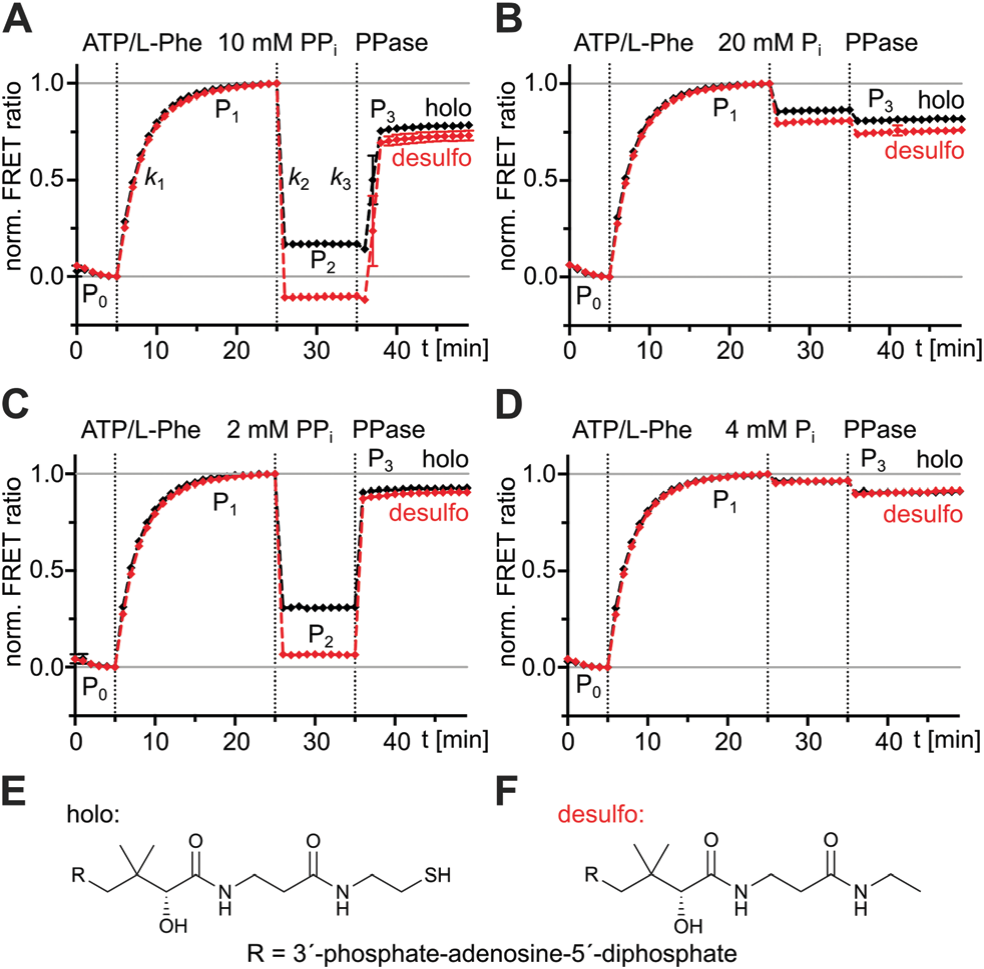
Reversible effect of excess PP_*i*_ on A-PCP sensors at the end of the aminoacylation pathway. Shown are time-courses of normalized FRET ratios of the holo- and desulfo-A-PCP sensors. To each protein (0.3 μM) was added ATP/l-Phe (2 mM each), PP_*i*_, P_*i*_ and PPase (0.2 U), respectively, as indicated. (A) and (B) PP_*i*_ at 10 mM and P_*i*_ at 20 mM, (C) and (D) PP_*i*_ at 2 mM and P_*i*_ at 4 mM. FRET signals were determined as ratios *I*_a_/*I*_d_ (intensity acceptor/intensity donor) and normalized between 0 and 1 using the signal at the time point before substrate addition and the respective maximum of the measurement series as reference points. The temporal resolution of our experimental setup only allowed to report minimal values for the high rate constants *k*_2_ and *k*_3_. Fitting of data show the mean ± SD of three (A and B) or two (C and D) independent experiments, each with three (A and B) or two (C and D) technical repeats. (E) and (F) Structure of coenzyme A and its desulfo analog used for the preparation of the indicated sensors.

We triggered the aminoacylation pathway by addition of the substrates ATP/l-Phe (2 mM each) and observed an increase of the FRET ratio from plateau P_0_ to plateau P_1_ with a rate constant *k*_1_ = (5.6 ± 0.3) × 10^−3^ s^−1^, consistent with previous measurements (Fig. 2A, black line; A-PCP sensor also termed holo sensor for its functional Ppant prosthetic group).^21^ The formation of the l-phenylalanyl thioester (l-Phe-Ppant) occurs at the same rate, as previously shown by mass spectrometry,^21^ suggesting that conformational changes to the transfer conformation coincide with aminoacylation. We confirmed that a desulfo variant of the A-PCP sensor, lacking the thiol group of the Ppant arm (Fig. 2E and F), follows the same *k*_1_ kinetics (Fig. 2A, red line; (5.3 ± 0.4) × 10^−3^ s^−1^), implying that progression to the transfer conformation, and not the chemical catalysis of the thiolation reaction itself, is rate-determining.^21^ Since the aminoacylated A-PCP didomain of the holo sensor can catalyze a second round of l-Phe-AMP formation for which it has to go through the O and A conformations, the observed shift to the transfer conformation suggests the latter to be in rapid equilibrium with the other conformations, yet to be the dominating species in the conformational mixture.^21^

Subsequent addition of excess PP_*i*_ (10 mM) results in a rapid drop of the FRET ratio to a stable plateau (P_2_), indicating the loss of the transfer conformation, at least for the most part (Fig. 2A). This change was previously rationalized by a conformational reversal of the A domain, induced by steric competition of PP_*i*_ with the side chain of R439 of the A8 loop that in the T conformation forms a salt bridge with E327 from the A^N^ subdomain. Loss of the T conformation would be accompanied by a loss of the PCP binding site on the A domain.^21^ The EC_50_ = 320 ± 50 μM of PP_*i*_ derived from a dose–response curve under these conditions^21^ is consistent with its influence on the high and low FRET conformations of the A-PCP sensor.

We then aimed to further study the reversibility of the PP_*i*_ effect. We added inorganic pyrophosphatase (PPase) to the equilibrium of plateau P_2_ to hydrolyze and remove all PP_*i*_. Indeed, we observed an increase of the FRET ratio to reach plateau P_3_, consistent with the idea of reversibility (Fig. 2A). It occurred shortly after addition of PPase, reflecting the time period the PPase required to hydrolyze the excess PP_*i*_. Interestingly, however, the rate constant *k*_3_ to reach plateau P_3_ with at least (4.8 ± 0.9) × 10^−2^ s^−1^ was much higher than the slow *k*_1_ (at least about 10-fold). The two different rates suggested that a different and previously undetected mechanism was at play to reach the high FRET conformational ensemble of P_3_ from P_2_ compared to the pathway from P_0_ to P_1_. We first hypothesized that the *k*_3_ rate constant could be explained by the l-Phe-Ppant thioester, which is present at plateau P_2_ but not at plateau P_0_, because the thioester should favor the adoption of the high FRET post-transfer conformation by enhancing the affinity of the PCP to the A domain. However, a similar experiment with the desulfo sensor, which allowed us to selectively sort out the influence of the l-Phe-Ppant thioester, showed a very similar and high *k*_3_ rate constant (Fig. 2A, red line; ≥(4.0 ± 0.8) × 10^−2^ s^−1^), indicating that the unknown mechanism at play when conformationally progressing from P_2_ to P_3_ must be independent of the l-Phe-Ppant thioester.

The observed lower level of plateau P_3_ compared to P_1_ turned out to be a result of the 20 mM orthophosphate (P_*i*_) formed through the hydrolysis of the 10 mM PP_*i*_ and the PPase itself, because their individual addition in control experiments led to the same decrease of the FRET ratio (Fig. 2B). Further control experiments with successive additions of ATP/l-Phe, then PP_*i*_ (2 mM) or P_*i*_ (4 mM) at lower concentrations, and then PPase, confirmed the dose-dependent effect of PP_*i*_ and resulted in nearly identical P_1_ and P_3_ plateau levels with unchanged kinetic rates (Fig. 2C and D). We also confirmed that the high P_*i*_ concentrations resulting from the PP_*i*_ hydrolysis did not affect the *k*_1_ rate constant (Fig. S2, ESI†). Thus, P_1_ and P_3_ represent the proteins in the same equilibrated conformations and the effects of PP_*i*_ addition and removal are fully reversible.

### Individual substrate binding is rapid and cannot account for a slow *k*_1_ rate

After having ruled out the formed l-Phe-Ppant thioester as the underlying cause for the high rate constant *k*_3_ compared to *k*_1_, we sought to understand why rate constant *k*_1_ was low. *k*_1_ describes the multistep aminoacylation pathway from the unliganded enzyme to the thioester-loaded form. To measure initial substrate binding, we used tryptophan fluorescence spectroscopy^30,31^ with the stand-alone A domain. Both ATP and l-Phe were found to bind very rapidly (≥(0.9 ± 1.2) × 10^−1^ s^−1^), excluding substrate binding as the cause for the slow *k*_1_ (Fig. S3, ESI†). This finding is consistent with previous kinetic analyses of substrate binding^32^ and the adenylation reaction. Both the initial single turn-over l-Phe-AMP formation of the wildtype (wt) protein^33^ as well as ATP/PP_*i*_ exchange reaction under steady-state conditions of the A-PCP sensor^21^ are significantly faster with rate constants ∼840-fold and ∼205-fold higher than *k*_1_, respectively. Thus, none of the chemical reactions and associated conformational changes up to the formation of the aminoacyl adenylate and the adoption of the A conformation can account for the slow step with the rate constant *k*_1_.

**Fig. 3:**
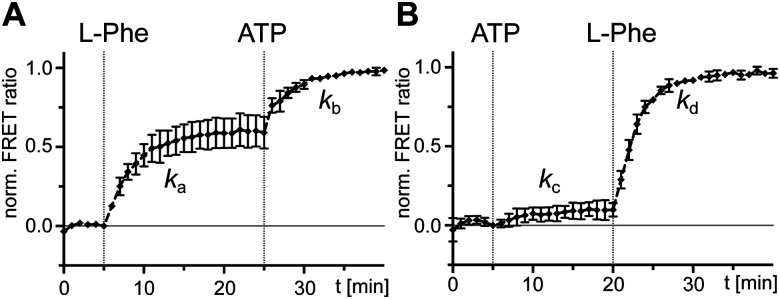
Probing separated addition of ATP and l-Phe with holo-A-PCP sensor. Shown are normalized time-courses of the FRET ratio. At the indicated time points, the holo-A-PCP sensor (0.3 μM) was exposed to (A) first l-Phe (2 mM) and then ATP (2 mM) or (B) first ATP (2 mM) and then l-Phe (2 mM). The rates of the marked increases of the FRET ratio were fitted to *k*_a_ = (4.5 ± 0.3) × 10^−3^ s^−1^, *k*_b_ = (5.7 ± 0.4) × 10^−3^ s^−1^, *k*_c_ = (3.5 ± 1.4) × 10^−3^ s^−1^ and *k*_d_ = (5.4 ± 0.1) × 10^−3^ s^−1^. FRET signals were determined as ratios *I*_a_/*I*_d_ (intensity acceptor/intensity donor) and normalized between 0 and 1 using the signal at the time point before substrate addition and the respective maximum of the measurement series as reference points. Shown are the mean ± SD of three independent measurements each consisting of three technical replicates.

We then investigated the effect of single substrates on conformational changes measured with the A-PCP sensor. Addition of l-Phe or ATP in individual form resulted in modest and minimal increases of the FRET ratio, respectively, suggesting partial conformational changes to the high FRET transfer conformation upon substrate binding, at least in case of l-Phe.^21^ Time-course experiments for these processes revealed a slow rate for the experiment with l-Phe, very similar to *k*_1_ (Fig. 3A and B). Subsequent addition of the second substrate in both possible orders triggers catalysis and led to further increases of the FRET ratio, again with rate constants comparable to *k*_1_ (Fig. 3A and B). These results further corroborated the idea that the slow step with the *k*_1_ rate constant is linked to a conformational change in the progression towards the transfer conformation.

### Proposal for a new conformational state, which is adopted after the rate-determining step

**Fig. 4:**
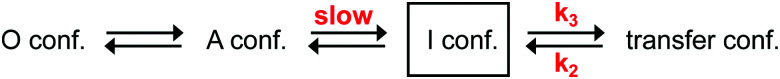
An expanded model of the domain alternation mechanism in the aminoacylation pathway of A-PCP didomain triggered by the addition of substrates. Note that the I conformation (I conf.) may represent more than one distinct conformation. In the O and A conformations, the PCP has no defined location relative to the A domain, whereas it binds the A domain in the transfer conformation with the latter being in T conformation.

Our above-described experiments and considerations revealed that the rate-determining slow step in the aminoacylation pathway of the A-PCP didomain is dependent on a conformational change, and not on enzymatic catalysis. It has to occur somewhere along the pathway to reach the pre-transfer conformation, which is the last conformation the holo- and the desulfo-A-PCP sensors have in common (Fig. 1 and 2). As a candidate for the slow conformational change we could rule out the binding of the PCP to the A domain with concomitant release of PP_*i*_ to give the productive transfer conformation, because this conformational change was found to occur with the high *k*_3_ rate constant following addition and subsequent removal of excess PP_*i*_ (Fig. 2A and C). Thus, the slow conformational change must occur *before* the fast adoption of the transfer conformation. As outlined above, it also has to occur *after* the fast adoption of the closed A conformation of the A domain for the rapid aminoacyl adenylate formation.^21,31^ Therefore, our data can only be explained with at least one new and unknown intermediary conformation (I conformation) that is present after the A conformation when the A domain is en route towards the T conformation (illustrated in Fig. 4). The adoption of this I conformation represents the rate-determining step of the aminoacylation pathway. The presence of more than one new I conformation cannot be ruled out.

The observed consequences of adding excess PP_*i*_ at the end of the aminoacylation pathway fit into this picture as a triggered conformational change in the reverse direction on the reaction coordinate. From the P_1_ level representing the enzyme significantly populating the transfer conformation, a population shift with the high *k*_2_ rate constant is triggered to a new equilibrium of conformations represented by the FRET-plateau P_2_ that may include one or more possible new I conformation(s). Upon removal of PP_*i*_ by PPase the conformational mixture is driven again in the forward direction of the reaction coordinate with the high *k*_*3*_ rate constant to equilibrate at FRET-plateau P_3_ that represents an increased population of the transfer conformation, similar to P_1_ when corrected for the presence of orthophosphate (see above, Fig. 2A and C).

### The l-Phe-Ppant thioester can be formed in the presence of high PP_*i*_ levels

**Fig. 5:**
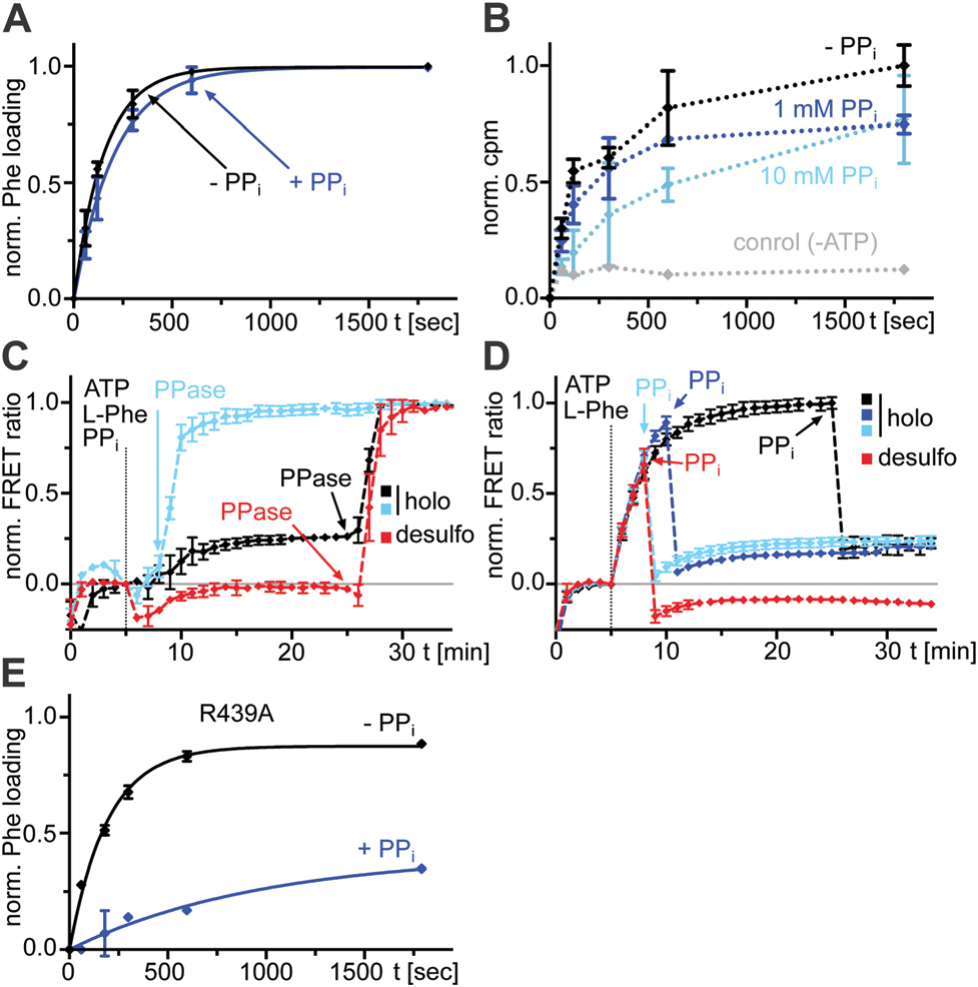
Effect of excess PP_*i*_ on the aminoacylation reaction of an A-PCP didomain. (A) Time-courses of l-Phe-Ppant thioester formation monitored by ESI-MS. Holo-A(ΔCys)-PCP-eGFP (10 μM) was mixed with ATP/l-Phe (each 2 mM) and PP_*i*_ (5 mM) as indicated. Shown are fitted curves of normalized data with the mean ± SD of three independent experiments. The rate constants are *k*_with PP_*i*__ = (4.8 ± 0.4) × 10^−3^ s^−1^ and *k*_without PP_*i*__ = (6.5 ± 0.8) × 10^−3^ s^−1^. (B) Time-course of [^3^H]Phe-Ppant thioester formation. Holo-A(ΔCys)-PCP-eGFP (0.5 μM) was mixed with ATP (1 mM) and l-Phe (4 μM total concentration, containing l-[3,4,5-^3^H]Phe). Shown are means ± SD from three independent experiments, each with three technical repeats. (C) and (D) Time-course FRET measurements of the aminoacylation reaction of the holo- and desulfo-A-PCP sensors (C) with PP_*i*_ and PPase or (D) only PP_*i*_ added at different time points. Each protein (0.3 μM) was mixed with ATP/l-Phe (each 2 mM). PP_*i*_ (10 mM) and PPase (0.2 U) were added at the indicated time points. FRET signals were determined as ratios *I*_a_/*I*_d_ (intensity acceptor/intensity donor) and normalized between 0 and 1 using the signal at the time point before substrate addition and the respective maximum of the measurement series as reference points. Data show the mean ± SD of three experiments with three technical repeats each. (E) Time-courses of l-Phe-Ppant thioester formation monitored by ESI-MS. The assay was performed as described in (A) using holo-A(ΔCys, R439A)-PCP-eGFP (10 μM) and with two independent experiments (*k*_without PP_*i*__ = (5.2 ± 0.3) × 10^−3^ s^−1^ and *k*_with PP_*i*__ = (1.0 ± 0.3) × 10^−3^ s^−1^).

We next investigated the effect of PP_*i*_ at excess concentrations on enzyme catalysis. Surprisingly, we could detect quantitative formation of the l-Phe-Ppant thioester when PP_*i*_ (5 or 10 mM) was added together with ATP/l-Phe at *t* = 0 min to holo-A-PCP-eGFP, using both mass spectrometry (MS) analysis (Fig. 5A) and a thiolation assay with radiolabeled l-Phe (Fig. 5B). The reaction occurred only at a slightly slower rate than in the absence of PP_*i*_. Consistent with these observations, the holo-A-PCP sensor revealed under these conditions an increase of the FRET ratio to reach a plateau corresponding to P_2_, with a rate constant of (2.9 ± 0.5) × 10^−3^ s^−1^, only modestly slower than *k*_1_ (Fig. 5C, black line). As previously discussed,^21^ the elevation of the P_2_ plateau compared to P_0_ is explained by partial rebinding of the formed l-Phe-Ppant thioester to the A domain to stabilize the high FRET transfer conformation. Given the aminoacylation activity in the presence of PP_*i*_, the same P_2_ plateau can thus also be reached with excess PP_*i*_ being present from the beginning of the aminoacylation reaction. Consistent with this finding, the addition of PPase at the stage of this P_2_ plateau led to a rapid jump of the FRET ratio, similar to the observation in Fig. 2A and C for P_3_ and rate constant *k*_3_ (Fig. 5C black line; note that due to normalization of the FRET ratios this plateau now appears at a ratio = 1.0). Fig. 5C and D show that one can switch between the PP_*i*_ and the PPase conditions at any time during the aminoacylation reaction. The desulfo-A-PCP sensor behaved accordingly, except that its P_2_ values were lower due to the inability of forming a thioester (Fig. 5C and D, red lines).

**Fig. 6:**
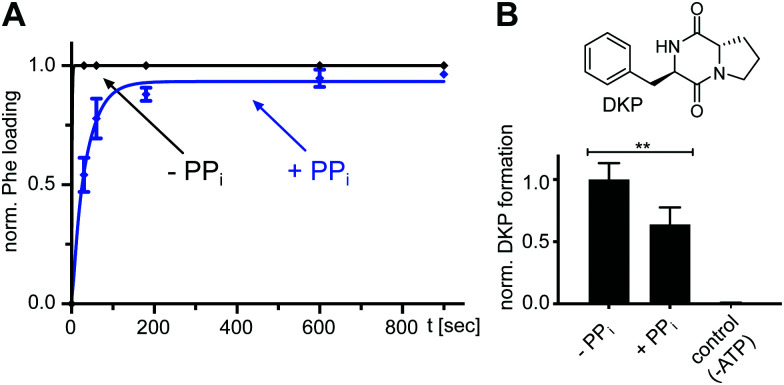
Effect of excess PP_*i*_ on the aminoacylation reaction of full-length wt NRPS modules. (A) Time-course of l-Phe-Ppant thioester formation of full-length holo-GrsA monitored by ESI-MS. GrsA (10 μM) was mixed with ATP/l-Phe (each 2 mM) and PP_*i*_ (5 mM) as indicated. Shown are fitted curves of normalized data with the mean ± SD of three independent experiments (*k*_with PP*i*_ = (2.9 ± 0.2) × 10^−2^ s^−1^). (B) Dipeptide product formation by full-length modules holo-GrsA and holo-TycB1. GrsA (0.5 μM) was mixed with TycB1 (5 μM), ATP (5 mM), l-Pro and l-Phe (each 2 mM), and PP_*i*_ (5 mM) as indicated. The formed d-Phe-l-Pro-diketopiperazine (DKP) was analyzed and quantified by HPLC. Shown are the mean ± SD of three independent replications ** indicates *p* ≤ 0.01, which was determined by Student's *t*-test.

We found that also the wildtype, full-length GrsA enzyme formed the aminoacyl-thioester in a virtually quantitative fashion in the presence of excess PP_*i*_ (Fig. 6A). Note that wt-GrsA is faster in the aminoacylation reaction than the A-PCP sensor, which is slowed down ∼17-fold by the removal of native cysteines.^21^ Furthermore, upon incubation of wt-GrsA with the module TycB1^34,35^ the dipeptide d-Phe-l-Pro was formed and accumulated as the cyclic diketopiperazine (DKP) through multiple turnovers (Fig. 6B).^34,35^ These results show that the aminoacylation activity in the presence of high PP_*i*_ concentrations is also observed for intact NRPSs or entire modules.

Specifically, these results suggested an unexpected population of the transfer conformation in the presence of ATP, l-Phe and excess PP_*i*_, at levels sufficient to support efficient catalysis of thioester formation. To test the robustness of this mechanism we sought to weaken the T conformation of the A domain. R439 from the A8 motif in the A^C^ domain forms a salt bridge with E327 from the A5 motif in the A^N^ domain as a key interaction to stabilize the T conformation. In the T conformation the R439 side chain occupies the same space as the β and γ phosphates of ATP in the A conformation and this space is likely similar to the binding site of PP_*i*_ following aminoacyl adenylate formation.^16,21,36^ We prepared an A-PCP didomain construct with the R439 side chain removed by mutagenesis. In the absence of excess PP_*i*_ and under saturating l-Phe concentrations, this A-PCP(R439A) mutant still formed the l-Phe-Ppant thioester with an almost unchanged rate. However, in the presence of 5 mM PP_*i*_ it was much stronger impaired than the unmutated protein (rate constant for thioester formation about 5-fold lower, Fig. 5E and Fig. S4, ESI†). These findings suggest that the R439 side chain provides an important contribution to ensure efficient directionality against the unfavorable equilibrium at high PP_*i*_ concentrations.

### Equilibrium HDX-MS analysis confirms mixture of conformational states and effect of PP_*i*_ on transfer conformation

**Fig. 7:**
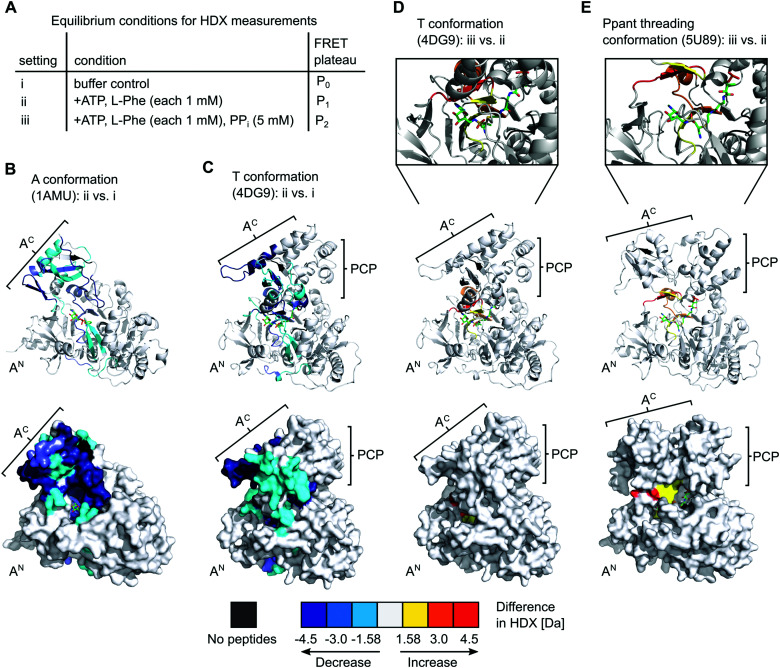
HDX-MS analysis of full-length holo-GrsA. The protein was pre-incubated for 30 min to reach equilibrium conditions before HDX-MS analysis was performed. (A) Overview of the three experimental settings. (B) to (E) Comparison of HD exchange rates between two different experimental settings illustrated by mapping the affected peptide stretches onto the crystal structure of the A domain (panel B)^37^ or of homologous A-PCP didomains (panels C, D and E).^19,39^ Each panel shows cartoon (top) and surface (bottom) representations of the same structure. The Ppant group trapped with an AMP-derived covalent inhibitor is depicted in stick representation (green). For (D) and (E) additional close-ups of the active-site are shown. The color legend at the bottom explains the gradual HDX response. For example, the red regions in (E) reflect an increased HD exchange rate at setting iii compared to setting ii. A^N^, A^C^, PCP (sub)domains and selected peptide stretches are indicated. Ligands and Ppant groups are represented by sticks. For representations of pdb files 4DG9 and 5U89, the sequence of GrsA A-PCP was modeled into the respective structures using Phyre2.^40^ Images were created with PyMol.

To gain further insight into the nature of the conformations induced by PP_*i*_ at high concentrations we turned to hydrogen–deuterium exchange mass spectrometry (HDX-MS). Using the wildtype, full-length holo-GrsA protein we analyzed three different experimental settings i to iii as shown in Fig. 7A, that represent the conditions of the three FRET ratio plateaus P_0_, P_1_ and P_2_, respectively. In HDX-MS, we identified a total of 336 peptides that covered 95% of the amino acid sequence of GrsA with 3.17-fold redundancy per amino acid (Dataset S1). Comparison between the three settings i to iii identified 13 sequence stretches that exhibited altered HD exchange rates, indicating significant conformational differences in GrsA under the corresponding conditions (Fig. S5–S8, ESI†). Nine of these sequence stretches, seq1 to seq9, are localized in either the A^N^ or the A^C^ subdomains and were relevant for the analysis of the interaction between A and PCP domains. All seq1 to seq9 exhibited a decreased deuterium incorporation, indicative of decreased solvent accessibility, in the presence of ATP and l-Phe (setting ii) compared to the buffer control (setting i) (Fig. S7A, ESI†). We mapped the sequence stretches onto crystal structures of the A domain and the A-PCP didomain in A^37^ and T conformation,^19,20^ respectively (Fig. 7B and C and Fig. S8, ESI†). We could attribute all the sequences to either regions of the protein involved in substrate binding or regions involved in domain–domain contacts between the A^N^ and A^C^ domains in one of the known closed conformations.^15^ Thus, these findings were in agreement with the current model of the A domain that generally adopts increased compactness upon addition of substrates,^38^ and specifically undergoes conformational changes from the open O conformation to the closed A, T and transfer conformations. Importantly, the fact that sequence stretches characteristic for each of the closed conformations were identified suggests that all these conformations were simultaneously present in a dynamic mixture of protein populations. For example, reduced HD exchange was observed for seq9 and seq6. Seq9 (aa508–530) of the A^C^ subdomain centers around the conserved K517 of the A10 motif, which interacts with the A^N^ subdomain in the A conformation. Seq6 (aa426–444) on the other hand harbors the conserved K434 on the opposite side of the A^C^ subdomain, the binding of which to the A^N^ is characteristic for the T and transfer conformations.

The additional presence of 5 mM PP_*i*_ (setting iii) also resulted in decreased HD exchange of seq1 to seq9 when compared to setting i of the buffer control (Fig. S7B, ESI†), hence also suggesting the presence of a mixture of the closed conformations. This finding further corroborates our observation that the entire aminoacylation pathway, including the thiolation reaction, can take place in the presence of PP_*i*_ at high concentrations. However, the shielding pattern was different when setting iii was compared to setting ii. Three stretches exhibited a relatively increased deuterium incorporation in the presence of PP_*i*_ (Fig. 7D and Fig. S7C, ESI†). These stretches, seq3, seq4 and seq6, are implicated in the A8 motif binding to the A^N^ domain, as discussed above, and in nucleotide binding (Fig. S6, ESI†). These findings suggest that, in the presence of PP_*i*_ (setting iii), the T and transfer conformations are relatively less populated in the equilibrium mixture of populations, possibly representing a partial opening of the A domain subunit from these conformations around the A8 motif. This interpretation is in agreement with our conclusions from the FRET experiments, namely that the addition of excess PP_*i*_ decreases the population levels of the transfer conformation, as manifested by the drop of the FRET ratio from plateau P_1_ to P_2_ (Fig. 2A and C).

Finally, sequence stretches seq10 and seq11 to seq13 (Fig. S6 and S7, ESI†) are located in the PCP and E domain, respectively, and exhibited elevated HD exchange in the presence of ATP/l-Phe (setting ii) compared to the buffer control (setting i), thus suggesting an altered conformation. This increase in HD exchange is partially diminished by the presence of PP_*i*_ (setting iii) implying PP_*i*_-dependent conformational alterations of these regions in the PCP and E domains as well. In fact, when mapping these regions on the structure of holo-PCP-E,^41^ the reciprocal changes in HD exchange of seq10–seq12 relative to those of seq1–seq9 would be consistent with a loosening of the PCP-E interaction upon increased PCP binding to the A domain, as expected.^42^ Further investigation will be required in the future to address the PCP-E interaction in detail.

## Discussion

In this study, we combined biochemical, FRET spectroscopic and HDX-MS analyses to investigate catalysis and conformational changes in the aminoacylation pathway of an A-PCP didomain. Our data implicate three key findings. First, at least one new conformational state must be inserted between the A and transfer conformations of the current domain alternation model (Fig. 1 and 4). The conversion from the A conformation to this postulated intermediate (I) conformation contains the rate-determining step of the aminoacylation pathway. Second, aminoacylation and NRP synthesis take place even in the presence of PP_*i*_ at high concentrations (up to 10 mM tested) and R439 in GrsA is important for this mechanism. Third, PP_*i*_ at such millimolar concentrations under aminoacylation conditions shifts the conformational mixture selectively from the equilibrium during and at the end of the aminoacylation pathway to a new mixture with relatively decreased populations of the T and transfer conformations. This latter conformational ensemble displays altered FRET characteristics of the A-PCP sensor (plateau P_2_ in Fig. 2A and C) and an altered HDX pattern (setting iii; Fig. 5). It can be rapidly reverted by removing PP_*i*_ with PPase.

**Fig. 8:**
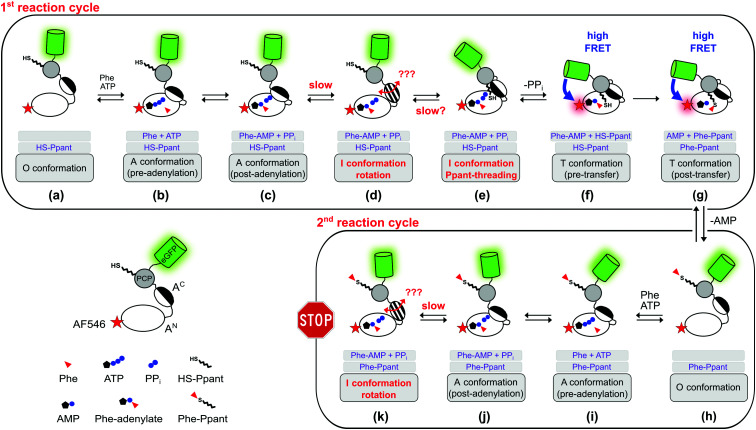
Expanded model of the conformational changes in the aminoacylation pathway of the holo-A-PCP sensor. Shown is their coupling to the chemical states. The model represents an expansion of our previous model.^21^ The three gray boxes below each protein illustration indicate (1) the substrates or ligands in the active site of the A domain, (2) the state of the Ppant prosthetic group, and (3) the designation of the illustrated conformation. The pre- (f) and post-transfer (g) conformations are high FRET states.^21^ Most, if not all, conformations are believed to be simultaneously populated in a protein ensemble and to be in dynamic equilibrium in individual proteins. Thus, an indicated conformation is not meant to constitute the only conformation the A-PCP protein adopts under the given conditions, but is rather to be seen as a conformation that is favored by a population shift at the respective chemical state compared to other stages in the pathway. Following the second adenylation cycle the Ppant-threading and transfer conformations cannot be adopted again because of competition of the l-Phe-AMP in the active site with the incoming l-Phe-Ppant thioester. Under product turnover conditions in the wildtype NRPS the aminoacylated Ppant-PCP can translocate to the E and C domains, which is not possible with the truncated A-PCP construct used in this study. Discharge at the C domain would likely be possible at all states from (h) to (k) and would start the next aminoacylation cycle. Note that the desulfo-A-PCP sensor can only access the limited set of chemical states (a) to (f). Addition of excess PP_*i*_ to either sensor is expected to disfavor conformational states (f) and (g), and to favor conformational states (d) and (e), and possibly non-transfer conformations with other ligand occupations as well. PP_*i*_ exchange with PP_*i*_ from outside the active site likely can occur at all stages that show PP_*i*_. Abbreviation: Phe is used instead of l-Phe in the figure for reasons of clarity.

In how far can the postulated and observed conformations be pinpointed with respect to their occurrence and order in the aminoacylation pathway? Fig. 8 summarizes the current knowledge of conformational changes in the aminoacylation pathway and our new proposals from this work, of which the ones relevant to this study will be explained in the following.

The revealed requirement for at least one additional I conformation on the way of the A-PCP didomain from the A to the transfer conformation is consistent with two simple biochemical and structural considerations: first, it is difficult to imagine how the A domain can switch between these two closed conformations without any partially or fully open intermediate. We propose an I conformation termed rotation conformation, in which the A^C^ subdomain is dissociated from the A^N^ part and thereby capable of rotation. Second, it appears entropically highly unlikely that PCP binding to the A domain involves threading the Ppant prosthetic group through a pre-formed narrow Ppant tunnel identified in crystal structures of the transfer conformation between the A^N^ and A^C^ subdomains.^18–20^ It seems more plausible to formulate an I conformation, here termed Ppant-threading conformation, in which the PCP interacts with the A domain in an open or half-open conformation, that allows the Ppant arm to be positioned towards the active site, and only with a final closing movement of the A^C^ and the PCP to adopt the transfer conformation. Fig. 8c–f shows the postulated insertion of the rotation and Ppant-threading conformations between the A and transfer conformations of the aminoacylation pathway.

Intriguingly, we realized that one deposited NRPS crystal structure (pdb 5U89) of unknown functional relevance fulfills our criteria for such a Ppant-threading conformation. Schmeing and co-workers reported the structure of an A-PCP-C fragment of *Bacillus subtilis* DhbF with the HS-Ppant moiety trapped by an adenosine-vinylsulfonamine inhibitor in the active site (Fig. 7E and Fig. S9A, ESI†).^39^ The A-PCP didomain in this ensemble shows the A^C^ subdomain rotated already so far towards the T conformation that the conserved lysine of the A8 motif (K434 in GrsA) is in the active site. However, the A^C^ subdomain is only partially closed down onto the A^N^ domain, with the salt bridge between E327 of the A^N^ and R439 of the A8 motif in A^C^ (GrsA numbering) not being formed. Importantly, in contrast to the narrow tunnel surrounding the Ppant arm in structures of the transfer conformation,^18–20^ the 5U89 structure therefore shows a cleft between the A^C^ and A^N^ subdomains, through which the prosthetic group could *enter* or *leave* the active site sideways without requiring additional domain movements (Fig. S9C, ESI†). Furthermore, the structure seems to be compatible with the presence of PP_*i*_ at high concentrations, because the distance between the residues corresponding to E327 and R439 created by the partial lift of the A^C^ from the A^N^ exactly forms a space and environment consistent with a PP_*i*_ binding site, allowing entry and release of PP_*i*_ (Fig. S10, ESI†). The presence of a PP_*i*_ binding site suggests that this structure could be induced by addition of PP_*i*_ at high concentrations (which represents the experimental condition of setting iii in our HDX-MS experiments, see Fig. 7). Further support for this structural assignment is provided by the striking consistency of our HDX data with the half-open conformation of the 5U89 structure. Fig. 7E shows the sequence stretches seq3, seq4 and seq6 of the GrsA A domain that became less shielded in the presence of PP_*i*_ (setting iii compared to setting ii). The localization of these stretches and their increased solvent accessibility nicely coincide with the partial lift of the A^C^ subdomain from the A^N^ subdomain relative to the T or transfer conformations. We therefore believe that the domain arrangement is a plausible blueprint for how Ppant can thread into the active site, although in our HDX experiments as well as in the observed P_2_ level of the FRET sensor (Fig. 2A and C) the A-PCP didomain is in the aminoacylated form and will therefore rather represent the conformational state when the Ppant arm leaves the active site.^39^ Finally, the PCP domain is somewhat translated and rotated in the 5U89 structure compared to the transfer conformation.^39^ This arrangement would likely result in a loss of the high FRET arrangement in our A-PCP sensor compared to the transfer conformation and thereby be consistent with the drop of the FRET ratio upon addition of PP_*i*_ (change from P_1_ to P_2_ in Fig. 2A and C).

Apart from these fitting structural considerations and assignments, it is not clear at this point which of the proposed conformations represents the new I conformation that we could postulate on basis of our kinetic analysis and which is adopted in the rate-determining step of the aminoacylation pathway. On the one hand, the domain association to form the Ppant-threading conformation could make this step rate-determining. On the other hand, adoption of the postulated rotation confirmation requires at least a partial dissociation of the A^C^ from the A^N^ subdomain with the bound reaction intermediates (l-Phe-AMP and PP_*i*_) in the closed A conformation and would therefore likely involve a significant energy barrier that would provide a rationale for why this conformational change is rate-determining.

Collectively, our experimental data together with biochemical considerations guided by the 5U89 structure can be integrated into a consistent model. We propose that after the adenylation reaction with the closed A conformation, the A^C^ subdomain partially or fully dissociates from the A^N^ to give a rotation I conformation. The A^C^ rotates and associates with the PCP to give a half-open Ppant-threading I conformation similar to the 5U89 structure, before finally adopting the closed didomain ensemble of the transfer conformation (Fig. 8c–f). Further work will be required to unambiguously assign the rate-determining step in this model. Importantly, our work also suggests that the in-solution techniques such as FRET and HDX are well suited to address the remaining questions on the NRPS biosynthetic machinery in the future.

## Methods

### General information

IPTG, ATP, HEPES and MgCl_2_ were purchased from AppliChem. TCEP was purchased from Carl Roth. Desulfo-coenzyme A and AMP were purchased from Jena Bioscience. AF546 maleimide and inorganic pyrophosphatase were purchased from Thermo Fisher Scientific. Ni-NTA agarose was purchased from Cube Biotech GmbH. Oligonucleotides were purchased from Biolegio. Other chemicals were purchased from AppliChem, Roth or Thermo Scientific. Experimental data were analyzed and fitted using GraphPad Prism 7.

### Cloning of expression plasmids

A(ΔCys) was prepared using restriction-free cloning^43^ using primer oFM92 (GTGGTGGTGGTGCTCGAGAAGCTTAGTCTACCCTCATCCCGAAAG) and oFM93 (CGCAGTTCGAAAAAGCTAGCGGTACCATGGTAAACAGTTCTAAAAGTATATTG) together with the plasmid coding for A(ΔCys)-PCP-eGFP as a template. Wildtype GrsA, A(ΔCys)-PCP-eGFP and A(ΔCys, R439A)-PCP-eGFP were cloned as previously described.^21^

### Gene overexpression and protein purification

Genes were expressed and the recombinant proteins purified as described before,^38^ either using the *Escherichia coli* Rosetta 2 (DE3) or the *E. coli* BL21 (DE3) strain. The concentration of all constructs after purification and dialysis was determined through their absorption at 280 nm, using the theoretical extinction coefficient estimated by ProtParam.^44^

### Chemical modification with AF546 maleimide

The single surface-exposed cysteine residue C152 of A(ΔCys)-PCP-eGFP was conjugated with AF546 maleimide as previously reported.^21^

### Post-translational conversion of apo into holo proteins

The post-translational modification with 4′-phosphopantetheine (Ppant) was carried out as described previously.^21^ Proteins (1–10 μM) were converted in their active holo-forms by addition of 4′-phosphopantetheinyl transferase Sfp (0.05 eq.), coenzyme A or desulfo coenzyme A (100 eq.) and TCEP (2 mM) in assay buffer (pH = 7.0; 50 mM HEPES, 100 mM NaCl, 1 mM EDTA, 10 mM MgCl_2_) and incubated overnight at 4 °C.

### FRET measurements

FRET measurements were performed using a quartz cuvette (Hellma) together with the SpectraMax M5 Multi-Mode Microplate Reader (Molecular Devices) or the FP-8500 Fluorescence Spectrometer (Jasco). As direct excitation wavelengths 470 nm was chosen for eGFP and 540 nm for AF546. The respective emission wavelengths were detected at 510 nm for eGFP and 570 nm for AF546. For the SpectraMax a filter with a 495 nm cutoff was applied, while for the FP-8500 a monochromator for both excitation as well as emission with a bandwidth of 5 nm was used. With this setup for each measurement the fluorescence of both eGFP and AF546 after direct excitation was recorded as well as the emission of AF546 after eGFP excitation (energy transfer). A-PCP sensors were used at a concentration of 300 nM in a total volume of 50 μL assay buffer (pH 7.0). The solution was incubated for 5 to 10 min until a stable fluorescence signal was recorded. At this point substrates or ligands were added as indicated in the figures (ATP and l-Phe at 2 mM; PP_*i*_ at 2, 5 or 10 mM; P_*i*_ at 4 or 20 mM). 0.2 U of PPase was added as indicated. Readings were taken every minute. The obtained raw data were normalized using the emission of AF546 after its direct excitation to eliminate fluorescence fluctuations based on varying enzyme concentration. Further, the recorded energy transfer was corrected for the donor bleed-through and the direct excitation of the acceptor fluorophore at 470 nm. Bleed-through was treated as a fraction of eGFP emission (8.16% for the SpectraMax and 5.89% for the FP-8500). The direct excitation of AF546 at 470 nm was calculated by the excitation of the standalone dye (SpectraMax)^21^ or determined as a fraction (6.48%) of the overall observed fluorescence of the dye after excitation at 540 nm.^45,46^ From the corrected data the FRET ratio was calculated (*I*_a_/*I*_d_) and normalized between 0 as the time point before substrate addition and 1 as the highest value obtained within one data set. Each FRET experiment was conducted with at least two independent measurements, each containing multiple technical repeats. The mean and SD of each set was calculated and the data pooled. The combined SDs (*S*_c_) were calculated using 
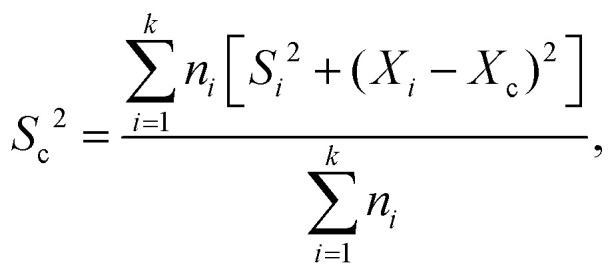
 with *X*_*i*_ and *X*_c_ representing the means of the individual and the combined data sets, respectively. Rate constants were fitted using a single exponential function assuming pseudo-first-order reactions.

### MS assay to detect the thioester formation

The basic principles of the assay were described earlier.^21^ The analysis was performed using an UltiMate™ 3000 RS system (Thermo Fisher Scientific GmbH) connected to a maXis II UHR-qTOF mass spectrometer (Bruker Daltonik GmbH) with a standard ESI source (Apollo, Bruker Daltonik GmbH). According to the protein concentration, an appropriate volume of the supernatant was loaded on a C4 column (Advance Bio RP-mAb C4, 2.1 mm × 50 mm, 3.5 μm, Agilent Technologies) at a flow rate of 0.6 mL min^−1^ in 5% eluent B (eluent A: 0.1% formic acid in water; eluent B: 0.1% formic acid in acetonitrile). After a desalting period of 7 minutes at 5% B, a steep gradient was applied (5–60% B in 2 min). MS settings: capillary voltage 4500 V, end-plate offset 500 V, nebulizer 5.0 bar, dry gas 9.0 L min^−1^, *T* = 200 °C, mass range *m*/*z* 300–3000. Data were analyzed with DataAnalysis 4.4 (Bruker Daltonik GmbH) and deconvolution was performed using the MaxEnt algorithm implemented in the software. According to Mann *et al.*,^47^ the deconvoluted height of multiply charged ions can be directly correlated to their abundance. Therefore, we used the area under the curve of the respective protein signals to determine their relative amount in the sample, assuming that the ionization efficiencies are comparable. The abundance of the loaded protein was plotted against time and fitted with a single exponential function.

### Determination of ATP and l-Phe binding by tryptophan fluorescence spectroscopy

The tryptophan fluorescence spectroscopy was carried out based on a previously described protocol.^30^ 500 nM of A(ΔCys) in assay buffer was incubated in a non-binding microplate (Greiner-Bio-One) for 10 min with varying concentrations of either ATP or l-Phe. Then, using a Tecan Infinite M1000 Pro microplate reader, the samples were excited at 290 nm (monochromator bandwidth 2.5 nm) and the fluorescence emissions at 330 nm (bandwidth of 5 nm) were recorded. The observed fluorescence change at 330 nm was plotted against the ligand concentration and the curve fitted with a binding equation with Hill slope. For the measurements of the binding kinetics 500 nM of A(ΔCys) were preincubated in assay buffer for 10 min before either ATP or l-Phe were added in a final concentration of 2 mM (total volume of 50 μL). Fluorescence readings at 330 nm after excitation at 290 nm were taken every minute, corrected for dilution effects and then normalized to the time point before the addition of the ligand. Buffer controls containing no protein did not display a change of fluorescence when ATP or l-Phe were added.

### L-[3,4,5-^3^H]Phe thioester formation assay

The assay was performed as previously described, either with or without additional PP_*i*_ as indicated.^21^ The raw data were normalized to the highest value of the sample containing no PP_*i*_ and fitted using a single exponential function.

### d-Phe-l-Pro-diketopiperazine (DKP) formation assay

This assay was basically performed as previously outlined.^34,35^ 5 μM of full-length holo-GrsA was incubated at 37 °C with 5 μM of the partner elongation module holo-TycB1 in assay buffer (pH 7.0) containing 1 mM l-Phe, 1 mM l-Pro and 10 mM MgCl_2_. With the addition of 5 mM ATP the reaction was started and after 30 min quenched with 400 μL *n*-butanol/chloroform (4 : 1 v/v) and 200 μL ddH_2_O. The organic phase was extracted and the watery phase washed with 400 μL of the organic mixture. The combined organic phase was then washed twice with 300 μL ddH_2_O. After each washing step the solutions were vortexed 20 sec and then centrifuged for 2 min (13 000 rpm, RT) to separate the phases. The resulting organic phase was dried at a vacuum concentrator and the remains resolved in 30 μL of HPLC starting conditions (95% ddH_2_O, 5% acetonitrile and 0.1% trifluoroacetic acid). The solution was analyzed by HPLC (reversed phase C18 column) and the area under the curve of the UV/Vis signal (210 nm) was used for the quantification of DKP.

### Hydrogen–deuterium exchange mass spectrometry (HDX-MS)

50 μM full-length holo-GrsA was incubated for 30 min at RT either without any ligand (setting i), in the presence of 1 mM ATP and 1 mM l-Phe (setting ii) or with 1 mM ATP, 1 mM l-Phe and 5 mM PP_*i*_ (setting iii). The following sample preparation and measurement was essentially performed as described previously.^48^ In brief, aided by a two-arm robotic autosampler (LEAP technologies), 7.5 μL of the reaction solution was mixed with 67.5 μL of D_2_O containing buffer to start H/D exchange. The buffer also contained the respective substrates to prevent their dilution. 55 μL of the reaction was mixed with an equal volume of quench buffer (400 mM KH_2_PO_4_/H_3_PO_4_, 2 M guanidine–HCl, pH 2.2) pre-chilled at 1 °C and 95 μL of the resulting mixture immediately injected into an ACQUITY UPLC M-Class System with HDX Technology (Waters Corporation).^49^ Undeuterated samples were prepared similarly by 10-fold dilution in H_2_O-containing buffer. Proteins were digested online with immobilized porcine pepsin at 12 °C with a constant flow (100 μL min^−1^) of water + 0.1% (v/v) formic acid, and the resulting peptic peptides collected on a trap column (2 mm × 2 cm) filled with POROS 20 R2 material (Thermo Scientific) kept at 0.5 °C. After 3 min, the trap column was placed in line with an ACQUITY UPLC BEH C18 1.7 μm 1.0 × 100 mm column (Waters Corporation), and the peptides eluted at 0.5 °C using a gradient of water + 0.1% (v/v) formic acid (A) and acetonitrile + 0.1% (v/v) formic acid (B) at 30 μL min^−1^ flow rate. Peptides were ionized by ESI (capillary temperature 250 °C, spray voltage 3.0 kV) and mass spectra acquired over a range of 50 to 2000 *m*/*z* on a Synapt G2-Si mass spectrometer with ion mobility separation (Waters Corporation) in enhanced high definition MS (HDMS^E^) or high definition MS (HDMS) modes for undeuterated and deuterated samples, respectively.^50,51^ During separation of the peptides, the pepsin column was washed three times with 80 μL of 4% (v/v) acetonitrile and 0.5 M guanidine hydrochloride. All measurements were carried out in triplicate. Peptides were identified and analyzed as described previously.^48^ For quantification of deuterium incorporation with DynamX 3.0 (Waters Corporation), peptides had to fulfil the following criteria: identification in 6 out of 9 undeuterated samples; minimum intensity of 25 000 counts; maximum length of 25 amino acids; minimum number of products of three; minimum number of products per amino acid of 0.1; maximum mass error of 25 ppm; retention time tolerance of 0.5 min. The difference sums of each peptide between two states (related to Fig. S6, S8 and S9, ESI†) were calculated by summing up the absolute difference in HDX between the two states at each time point ± their pooled standard deviation (*n* = 3 replicates). The 95% confidence interval as a significance threshold for the differences between two states was calculated as described before.^52^

## Author contributions

H. D. M. and H. Y. conceived the study. F. M., J. A., X. S., H. Y. and H. D. M. designed the experiments. F. M., A.-L. F., J. A. and W. S. performed the experiments. All authors analyzed and interpreted the results. H. D. M. and F. M. wrote the manuscript. All authors have given approval to the final version of the manuscript.

## Abbreviations

A conformation: Adenylation conformation
A domain: Adenylation domain
A^N^: Large N-terminal A subdomain
A^C^: Small C-terminal A subdomain
AF546: Alexa Fluor™ 546
A-PCP sensor: FRET sensor consisting of the A-PCP didomain
DKP: Diketopiperazine
eGFP: Enhanced green fluorescent protein
FRET: Förster resonance energy transfer
HDX-MS: Hydrogen–deuterium exchange mass spectrometry
I conformation: Intermediary conformation
O conformation: Open conformation
PCP: Peptidyl-carrier protein
Ppant: 4′-Phosphopantetheine
T-conformation: Thiolation conformation.

## Conflicts of interest

There are no conflicts of interest to declare.

## Supplementary Material

CB-002-D0CB00220H-s001

CB-002-D0CB00220H-s002

## References

[cit1] Süssmuth R. D., Mainz A. (2017). Angew. Chem., Int. Ed..

[cit2] Marahiel M. A., Stachelhaus T., Mootz H. D. (1997). Chem. Rev..

[cit3] Hur G. H., Vickery C. R., Burkart M. D. (2012). Nat. Prod. Rep..

[cit4] Bozhuyuk K. A. J., Linck A., Tietze A., Kranz J., Wesche F., Nowak S., Fleischhacker F., Shi Y. N., Grun P., Bode H. B. (2019). Nat. Chem..

[cit5] Calcott M. J., Owen J. G., Ackerley D. F. (2020). Nat. Commun..

[cit6] Mootz H. D., Schwarzer D., Marahiel M. A. (2002). ChemBioChem.

[cit7] Marahiel M. A. (2015). Nat. Prod. Rep..

[cit8] Stein T., Vater J., Kruft V., Otto A., Wittmann-Liebold B., Franke P., Panico M., McDowell R., Morris H. R. (1996). J. Biol. Chem..

[cit9] Cane D. E., Walsh C. T., Khosla C. (1998). Science.

[cit10] Reimer J. M., Haque A. S., Tarry M. J., Schmeing T. M. (2018). Curr. Opin. Struct. Biol..

[cit11] Weissman K. J. (2015). Nat. Chem. Biol..

[cit12] Tanovic A., Samel S. A., Essen L. O., Marahiel M. A. (2008). Science.

[cit13] Gulick A. M. (2016). Curr. Opin. Chem. Biol..

[cit14] Izore T., Cryle M. J. (2018). Nat. Prod. Rep..

[cit15] Gulick A. M. (2009). ACS Chem. Biol..

[cit16] Yonus H., Neumann P., Zimmermann S., May J. J., Marahiel M. A., Stubbs M. T. (2008). J. Biol. Chem..

[cit17] Wu R., Reger A. S., Lu X., Gulick A. M., Dunaway-Mariano D. (2009). Biochemistry.

[cit18] Drake E. J., Miller B. R., Shi C., Tarrasch J. T., Sundlov J. A., Allen C. L., Skiniotis G., Aldrich C. C., Gulick A. M. (2016). Nature.

[cit19] Mitchell C. A., Shi C., Aldrich C. C., Gulick A. M. (2012). Biochemistry.

[cit20] Sundlov J. A., Shi C., Wilson D. J., Aldrich C. C., Gulick A. M. (2012). Chem. Biol..

[cit21] Alfermann J., Sun X., Mayerthaler F., Morrell T. E., Dehling E., Volkmann G., Komatsuzaki T., Yang H., Mootz H. D. (2017). Nat. Chem. Biol..

[cit22] Reimer J. M., Aloise M. N., Harrison P. M., Schmeing T. M. (2016). Nature.

[cit23] Reimer J. M., Eivaskhani M., Harb I., Guarne A., Weigt M., Schmeing T. M. (2019). Science.

[cit24] Mijakovic I., Poncet S., Galinier A., Monedero V., Fieulaine S., Janin J., Nessler S., Marquez J. A., Scheffzek K., Hasenbein S., Hengstenberg W., Deutscher J. (2002). Proc. Natl. Acad. Sci. U. S. A..

[cit25] Chen J., Brevet A., Fromant M., Leveque F., Schmitter J. M., Blanquet S., Plateau P. (1990). J. Bacteriol..

[cit26] Kukko-Kalske E., Lintunen M., Inen M. K., Lahti R., Heinonen J. (1989). J. Bacteriol..

[cit27] Gevers W., Kleinkauf H., Lipmann F. (1968). Proc. Natl. Acad. Sci. U. S. A..

[cit28] Yamada M., Kurahashi K. (1969). J. Biochem..

[cit29] Vandamme E. J., Demain A. L. (1976). Antimicrob. Agents Chemother..

[cit30] Luo L., Burkart M. D., Stachelhaus T., Walsh C. T. (2001). J. Am. Chem. Soc..

[cit31] Sun X., Li H., Alfermann J., Mootz H. D., Yang H. (2014). Biochemistry.

[cit32] Stevens B. W., Lilien R. H., Georgiev I., Donald B. R., Anderson A. C. (2006). Biochemistry.

[cit33] Luo L., Walsh C. T. (2001). Biochemistry.

[cit34] Stachelhaus T., Mootz H. D., Bergendahl V., Marahiel M. A. (1998). J. Biol. Chem..

[cit35] Dehling E., Volkmann G., Matern J. C., Dorner W., Alfermann J., Diecker J., Mootz H. D. (2016). J. Mol. Biol..

[cit36] Kochan G., Pilka E. S., von Delft F., Oppermann U., Yue W. W. (2009). J. Mol. Biol..

[cit37] Conti E., Stachelhaus T., Marahiel M. A., Brick P. (1997). EMBO J..

[cit38] Zettler J., Mootz H. D. (2010). FEBS J..

[cit39] Tarry M. J., Haque A. S., Bui K. H., Schmeing T. M. (2017). Structure.

[cit40] Kelley L. A., Mezulis S., Yates C. M., Wass M. N., Sternberg M. J. (2015). Nat. Protoc..

[cit41] Chen W. H., Li K., Guntaka N. S., Bruner S. D. (2016). ACS Chem. Biol..

[cit42] Dehling E., Rüschenbaum J., Diecker J., Dörner W., Mootz H. D. (2020). Chem. Sci..

[cit43] van den Ent F., Lowe J. (2006). J. Biochem. Biophys. Methods.

[cit44] Gasteiger E., Hoogland C., Gattiker A., Duvaud S., Wilkins M. R., Appel R. D. and Bairoch A., in The Proteomics Protocols Handbook, ed. J. M. Walker, Humana Press, 2005 10.1385/1-59259-890-0:571

[cit45] Clegg R. M. (1992). Methods Enzymol..

[cit46] Kraynov V. S., Chamberlain C., Bokoch G. M., Schwartz M. A., Slabaugh S., Hahn K. M. (2000). Science.

[cit47] Mann M., Meng C. K., Fenn J. B. (1989). Anal. Chem..

[cit48] Osorio-Valeriano M., Altegoer F., Steinchen W., Urban S., Liu Y., Bange G., Thanbichler M. (2019). Cell.

[cit49] Wales T. E., Fadgen K. E., Gerhardt G. C., Engen J. R. (2008). Anal. Chem..

[cit50] Geromanos S. J., Vissers J. P., Silva J. C., Dorschel C. A., Li G. Z., Gorenstein M. V., Bateman R. H., Langridge J. I. (2009). Proteomics.

[cit51] Li G. Z., Vissers J. P., Silva J. C., Golick D., Gorenstein M. V., Geromanos S. J. (2009). Proteomics.

[cit52] Nielsen A. K., Moller I. R., Wang Y., Rasmussen S. G. F., Lindorff-Larsen K., Rand K. D., Loland C. J. (2019). Nat. Commun..

