# Supplementary material for: Intermediary conformations linked to the directionality of the aminoacylation pathway of nonribosomal peptide synthetases
Source: RSC Chem Biol. 2021 Mar 4;2(3):843–54. doi: 10.1039/d0cb00220h (PMC8341999; doi:10.1039/d0cb00220h)
Supplement: CB-002-D0CB00220H-s001 [file CB-002-D0CB00220H-s001.pdf]

Electronic Supplementary Material (ESI) for RSC Chemical Biology.
This journal is © The Royal Society of Chemistry 2021

Electronic Supplementary Information (ESI)

Intermediary conformations linked to the directionality of the aminoacylation pathway of nonribosomal peptide synthetases

Florian Mayerthaler[†], Anna-Lena Feldberg[†], Jonas Alfermann[†], Xun Sun[#,§], Wieland Steinchen[‡], Haw Yang[#], Henning D. Mootz[†,*]

[†]Institute of Biochemistry, Department of Chemistry and Pharmacy, University of Muenster, Münster, Germany

[#]Department of Chemistry, Princeton University, Princeton, New Jersey, USA

[‡]SYNMIKRO Research Center & Faculty of Chemistry, Philipps-University Marburg, Germany

* corresponding author, E-mail: Henning.Mootz@uni-muenster.de

This PDF file includes:

Supplementary Figures S1 to S11

Legend for Datasets S1

Supplementary References

Other supplementary materials for this manuscript include the following:

Datasets S1

Supplementary Figures

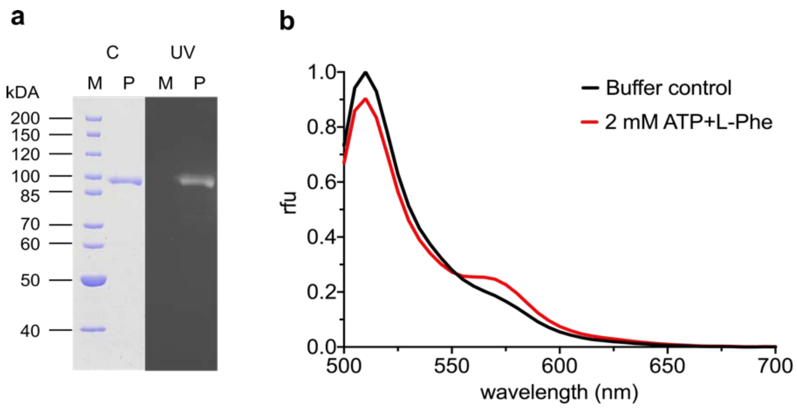

Supplementary Fig. 1 Preparation of the A-PCP sensor. (**A**) SDS–PAGE of purified apo-A(ΔCys)-PCP-eGFP after labeling of N152C with AF546 to yield the A-PCP sensor. C = Coomassie stain; UV = UV illumination of the same gel. (**B**) Representative fluorescence emission spectra of the holo-A-PCP sensor.

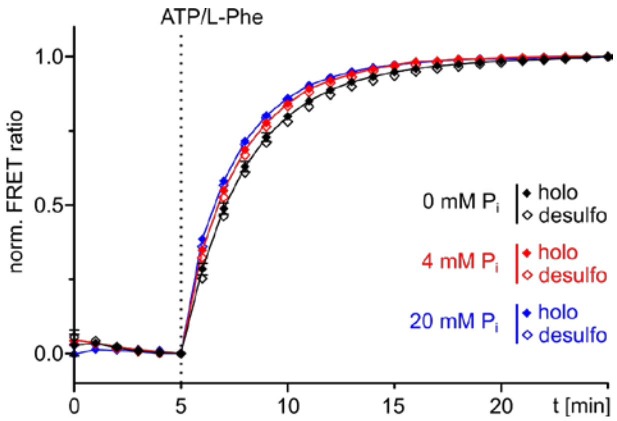

Supplementary Fig. 2 Effect of P_i on aminoacylation reactions monitored with A-PCP FRET sensors. Shown are time-courses of normalized FRET ratios of the holo- and desulfo-A-PCP sensors under the three indicated P_i concentrations. To each protein (0.3 µM) P_i was added from the beginning of the measurement and then ATP/L-Phe (2 mM each) as indicated. The curves for 0 mM P_i were taken from Figure 2 in the main text for comparison. FRET signals were determined as ratios I_a/I_d (intensity acceptor /intensity donor) and normalized between 0 and 1 using the signal at the time point before ATP/L-Phe addition and the respective maximum of the measurement series as reference points. Fitting revealed rate constants for 4 mM P_i ($k_{holo} = (6.5 \pm 0.1) \times 10^{-3}\ s^{-1}$ and $k_{desulfo} = (6.2 \pm 0.04) \times 10^{-3}\ s^{-1}$) and 20 mM P_i ($k_{holo} = (7.2 \pm 0.1) \times 10^{-3}\ s^{-1}$ and $k_{holo} = (6.9 \pm 0.1) \times 10^{-3}\ s^{-1}$). Data show the mean ± SD of two independent experiments, each with two technical repeats.

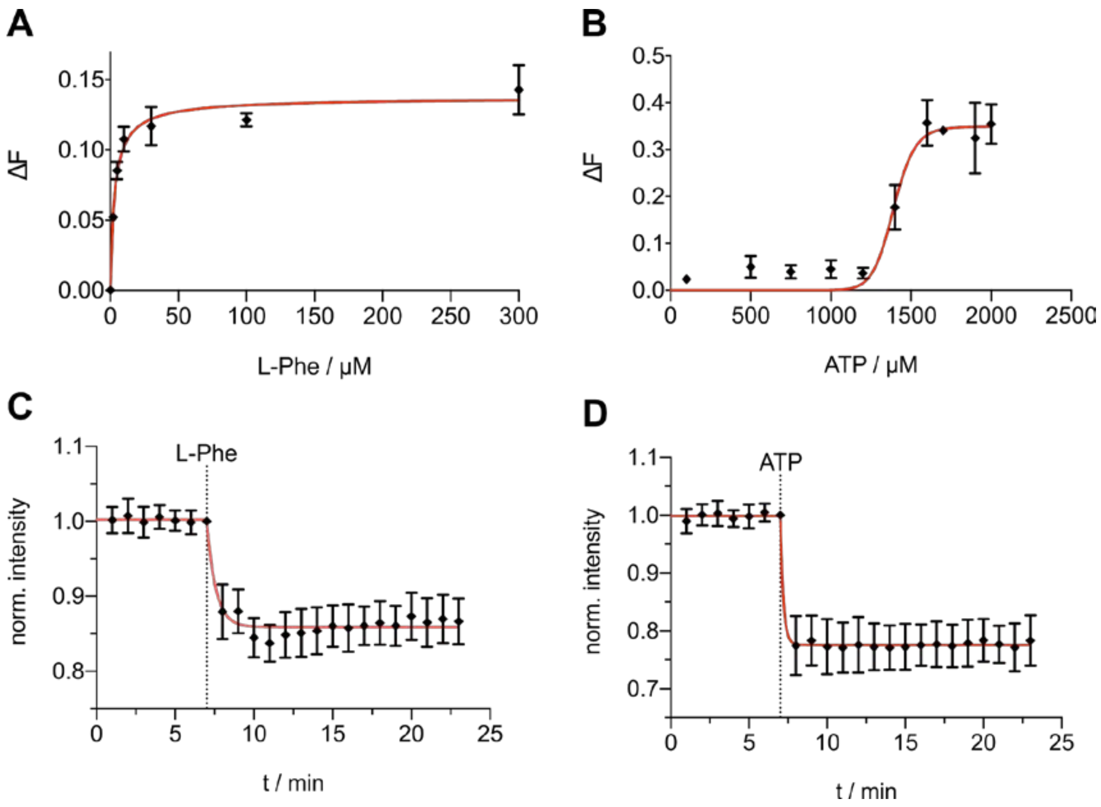

Supplementary Fig. 3 Detection of ATP and L-Phe binding to A(ΔCys) using tryptophan fluorescence spectroscopy. (**A**) For the determination of the dissociation constant of L-Phe, 0.5 µM of the enzyme were mixed with the indicated concentrations of L-Phe, incubated for 30 min at 25 °C and the fluorescence recorded. A K_D value of 3.1 ± 0.7 µM was determined, consistent with previously reported results.[1] (**B**) Similar experiment as in (**A**) to determine the dissociation constant of ATP (K_D = 1.39 ± 0.03 mM). (**C**) and (**D**) Time-resolved tryptophan fluorescence measurements to dissect the binding kinetic of L-Phe (**C**) and ATP (**D**). 0.5 µM of the enzyme were pre-incubated for 10 min at 25 °C at which point 2 mM of either substrate was added. Data display the mean ± SD of three (**A**, **B**) or ten (**C**, **D**) measurements. For (**A**) and (**B**) the K_D was calculated using the five parameters logistic regression.[2]

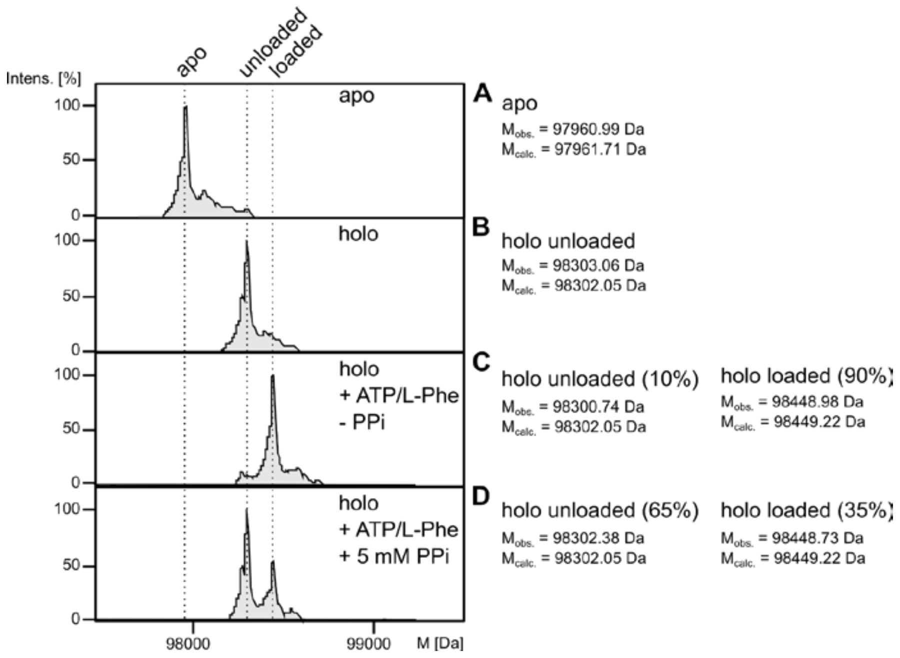

Supplementary Fig. 4 MS-analysis of the thioester formation using the holo-A(ΔCys, R439A)-PCP-eGFP construct. Panel (**A**) displays the apo protein and panel (**B**) the holo protein after the Ppant modification. In panels (**C**) and (**D**) 10 µM of the protein were mixed with 2 mM of each ATP and L-Phe, without (**C**) or with 5 mM PP_i (**D**) as indicated. After incubation for 30 min the samples were mixed with 1% FA and subjected to MS measurements. Exemplary data of one measurement is shown.

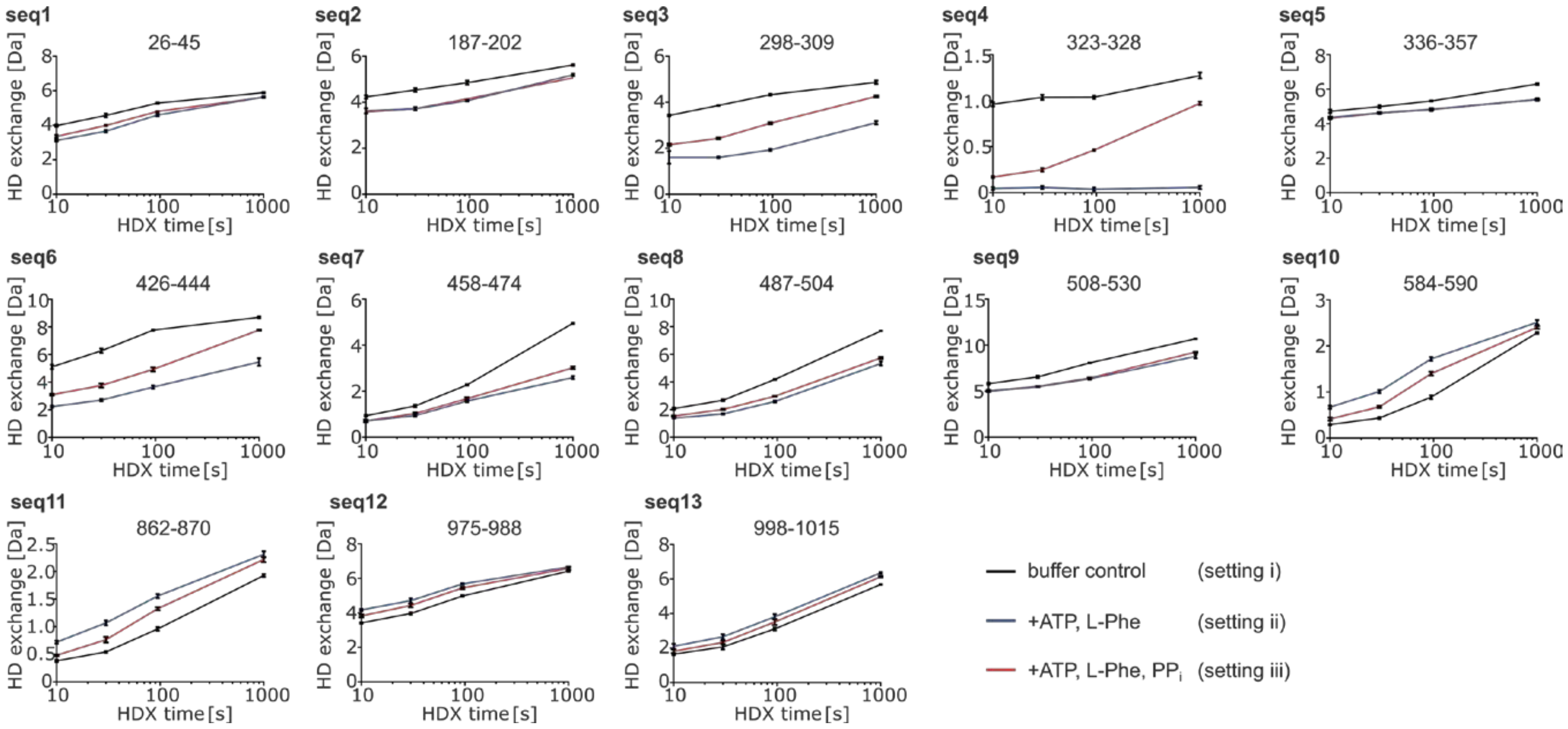

Supplementary Fig. 5 Hydrogen/deuterium (HD) exchange curves of 13 representative sequence stretches of full-length holo-GrsA. Those peptides displayed under incubation with ATP, L-Phe (blue, setting ii) or with ATP, L-Phe, PPi (red, setting iii) altered rates compared to the buffer control (black, setting i). The numbers above the plot represent the order of their appearance in GrsA (seq1 to seq13) and their respective amino acid range. Data represent the mean ± SD of three technical replicates.

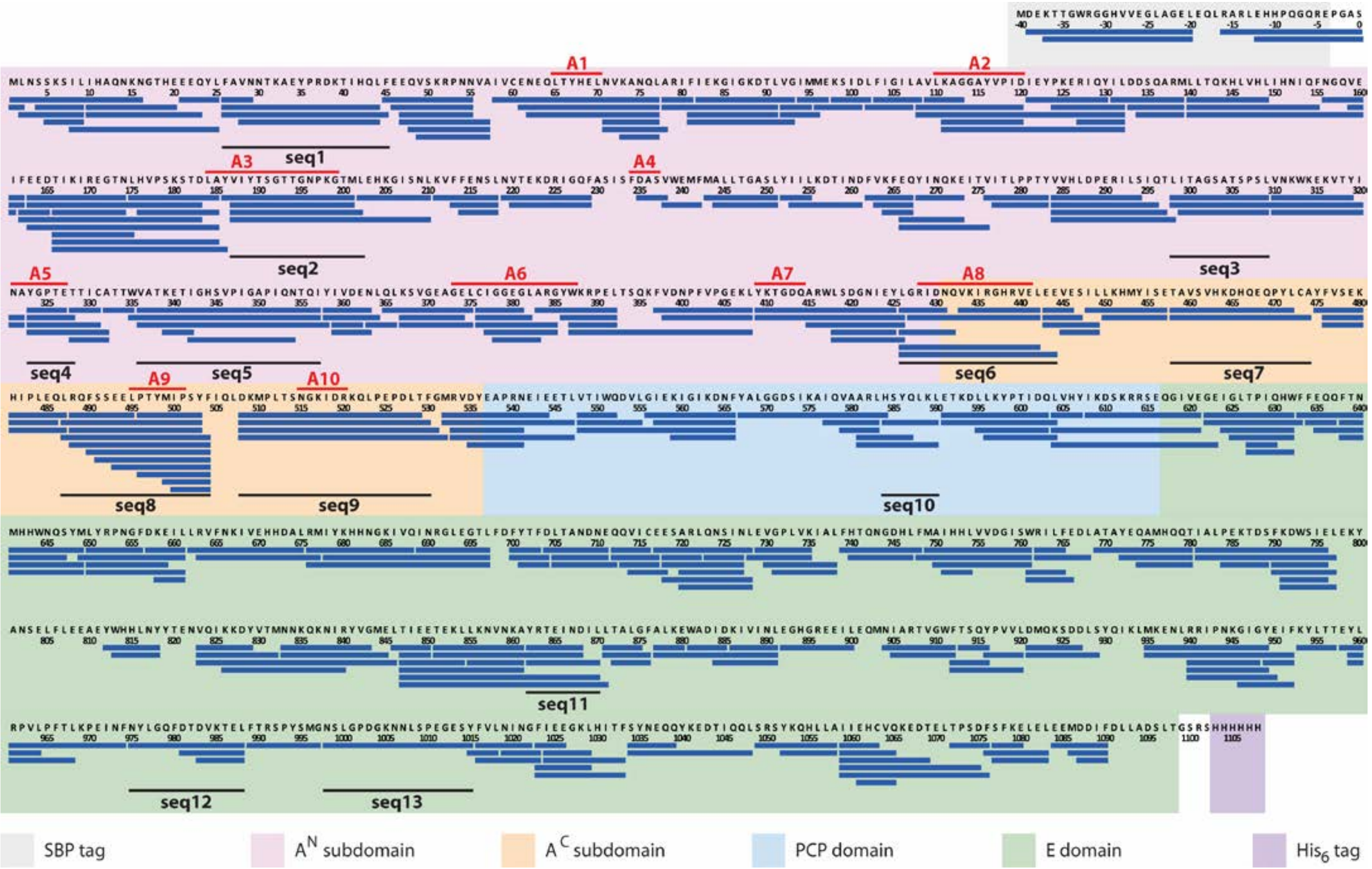

Supplementary Fig. 6 HDX-MS peptide coverage of full-length holo-GrsA displayed on the amino acid sequence of the protein. The numbering is according to Uniprot-ID: P0C062. Blue bars represent peptides that were identified and analyzed for their HD exchange. The locations of 13 peptide stretches (seq1 to seq13) that showed altered HD exchange under the different experimental settings i, ii and iii are shown as black bars. The core motifs A1-A10 in the A domain according to Marahiel *et al* [3] are highlighted in red. The color code of the background refers to the different domains and tags of the protein. Uncolored areas are inserted non-functional linker regions.

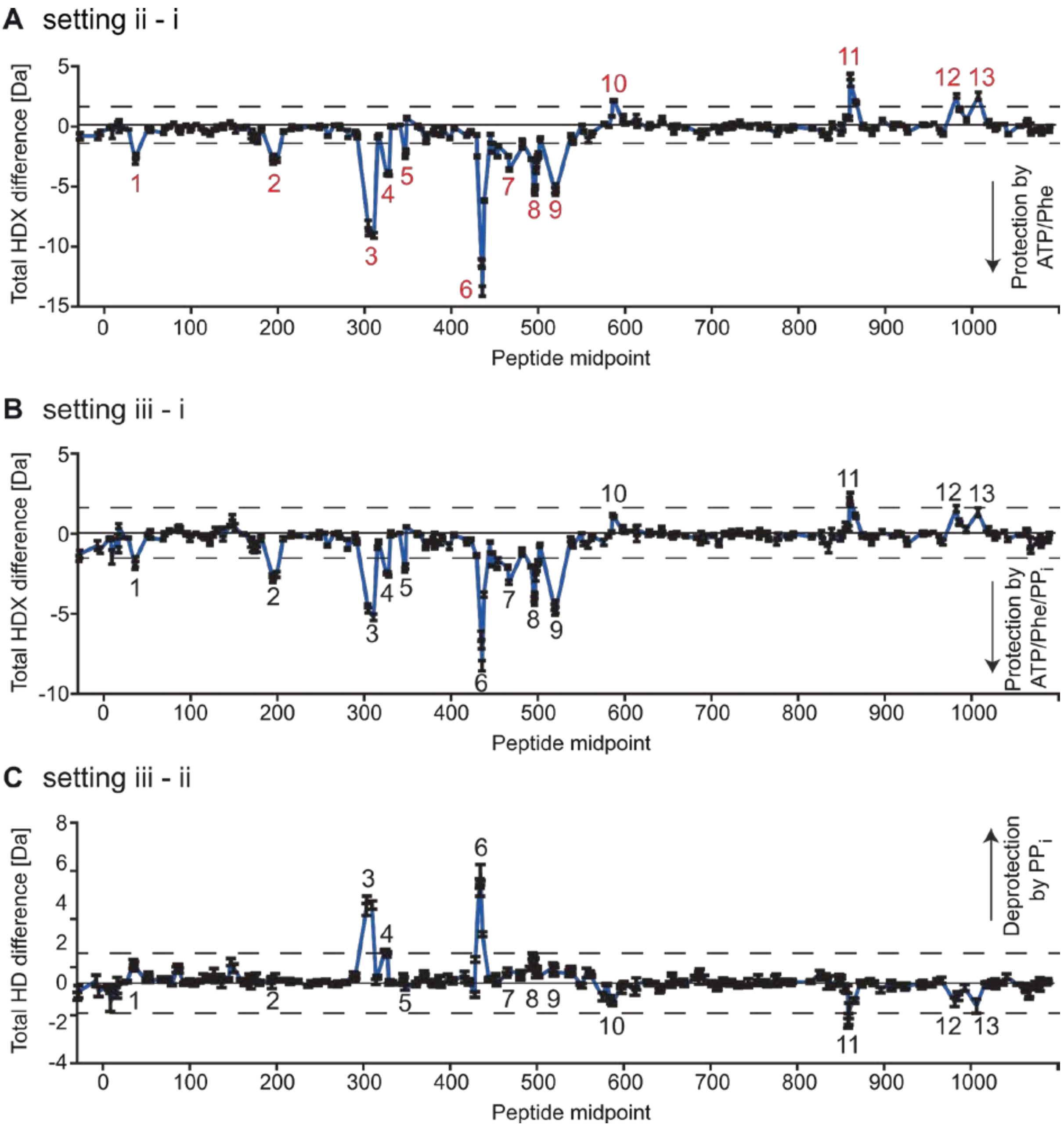

Supplementary Fig. 7 Differences in hydrogen-deuterium exchange (HDX) of holo-GrsA between the three settings: buffer control (i), ATP + L-Phe (ii) and ATP + L-Phe + PP$_i$ (iii). (**A**) depicts the difference of setting ii minus setting i, (**B**) depicts the difference of setting iii minus i and (**C**) depicts the difference of setting iii minus ii. To yield the absolute HDX change, the differences in HDX between settings i, ii and iii were calculated for each time point (i.e. 10, 30, 95, 1,000 s) and then summed up. The graphs depict the means ± SD (n = 3) of the total HDX difference according to the midpoint of the peptides (calculated as sum of the number of the first and last amino acid of the peptide divided by two). The dashed lines indicate the limits of the 95% confidence interval. Numbers in bold indicate statistical significance. Numbers above the plot denote the peptides shown in Figs. S6 and S7 with peptides exhibiting significantly increased or decreased HDX colored in red and blue, respectively.

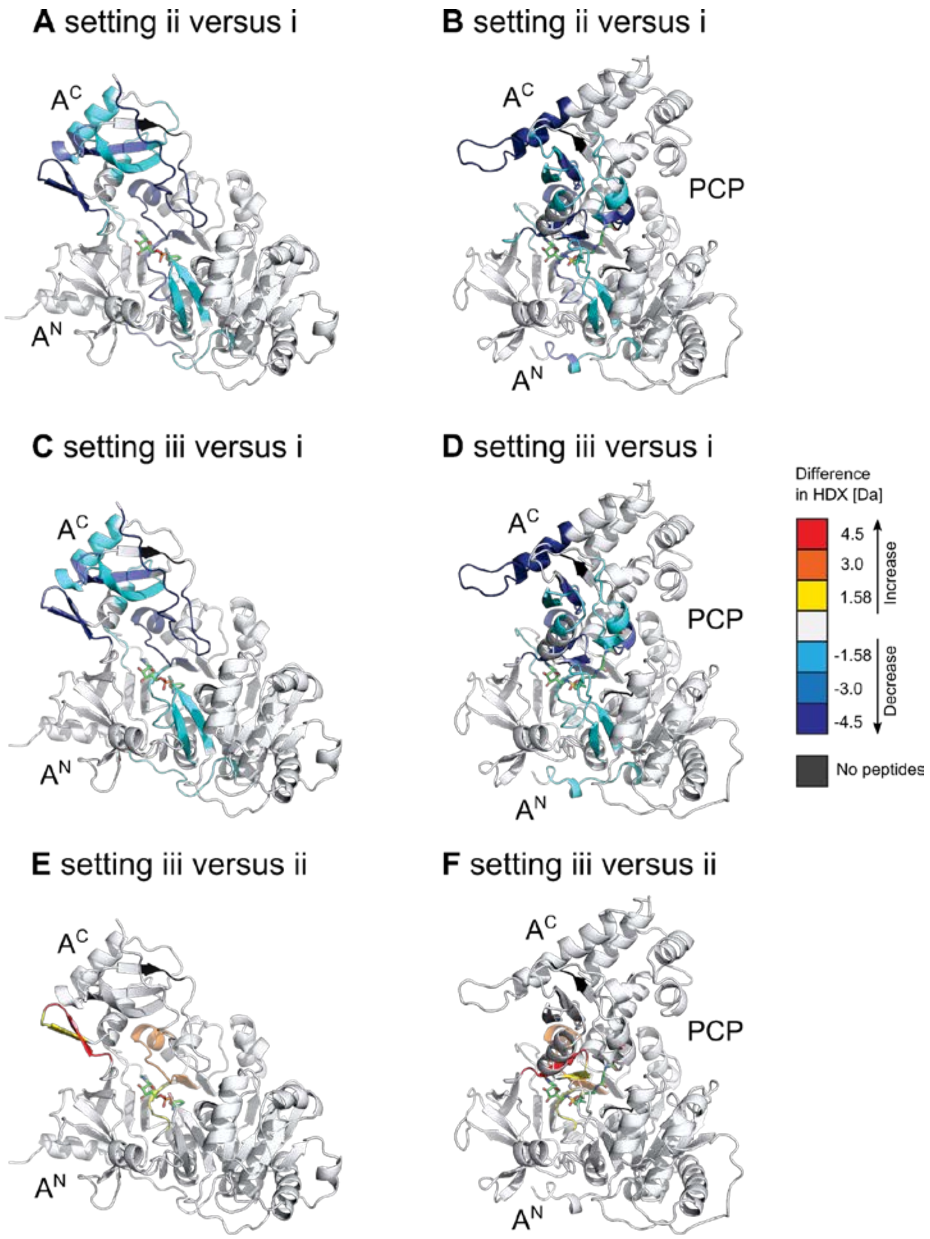

Supplementary Fig. 8 Regions of the A-PCP didomain in holo-GrsA with altered H/D exchange under settings (ii) and (iii) relative to (i) mapped onto crystal structures representing either the A (panels A, C and E) or the T conformation (panels B, D and F). (**A**) and (**B**) compare settings (i) to (ii), (**C**) and (**D**) compare (i) to (iii), and (**E**) and (**F**) compare (ii) to (iii). The structures are colored according to the HDX differences between the two conditions. The color legend at the bottom explains the gradual HDX response. In cases of partially overlapping peptides with disparate HDX behavior (i.e., only one of two partially overlapping peptides exhibiting a significant difference in HDX), the border for coloring was set in between the shared peptide stretch. The Ppant group trapped with an AMP-derived covalent inhibitor is depicted in stick representation (green). The structure of the A conformation is based on pdb 1AMU[4] and the one of the T conformation on pdb 4DG9[5] modeled to the GrsA sequence with Phyre2 (Ref[6]).

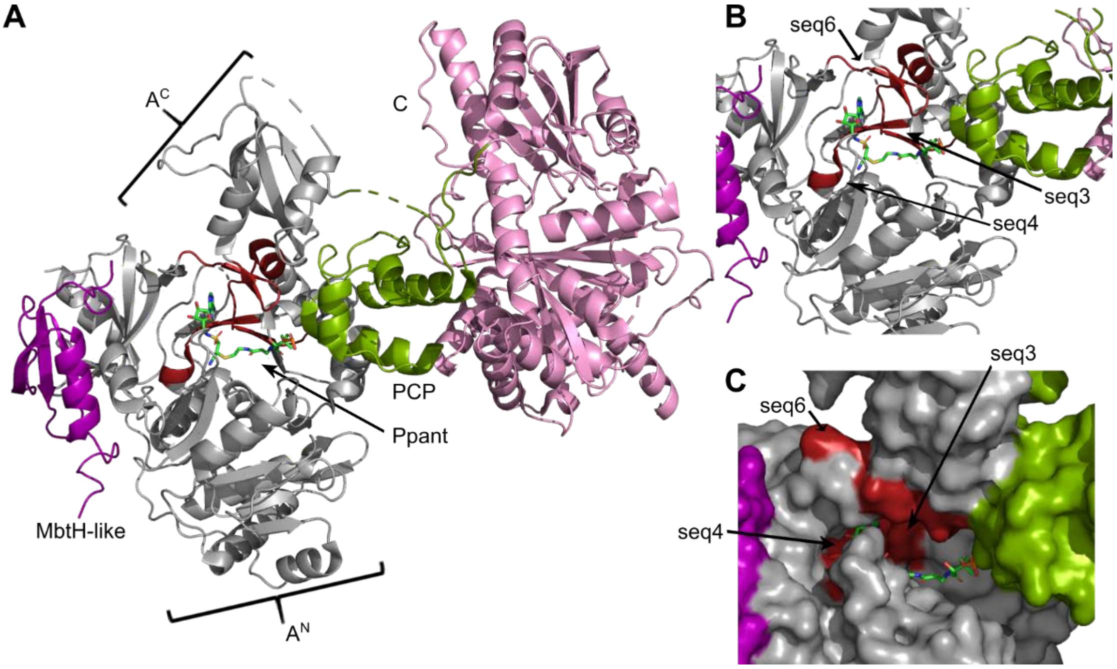

Supplementary Fig. 9 Previously reported crystal structure (pdb 5U89)[7] that appears fitting to represent the proposed Ppant-threading I conformation (**A**) Structure of the DhbF NRPS construct composed of A, PCP and C domains spanning across two modules and an MbtH-like protein bound to the A domain.[7] The structure represents our proposed Ppant-threading I conformation in which A^N and A^C (both grey) are partially detached from each other in a half-open state while the PCP-domain (green) with its Ppant group (colored based on elements) is bound with the Ppant pointing into the active site, where it is covalently captured with a vinylsulfone inhibitor.[7] The red colored areas inside the A domain refer to sequence stretches seq3, seq4 and seq6 that showed in the HDX-MS experiments higher H/D exchange rates when comparing setting (iii) to setting (ii) (Figs. S5 and S7). The C domain is shown in pink and the MbtH-like protein in purple. (**B**) Zoom-in on the active site of the A domain. (**C**) Surface representation of the structure as shown in (**B**), which highlights the cleft between A^N and A^C that might serve for the Ppant group to enter and exit the active site.

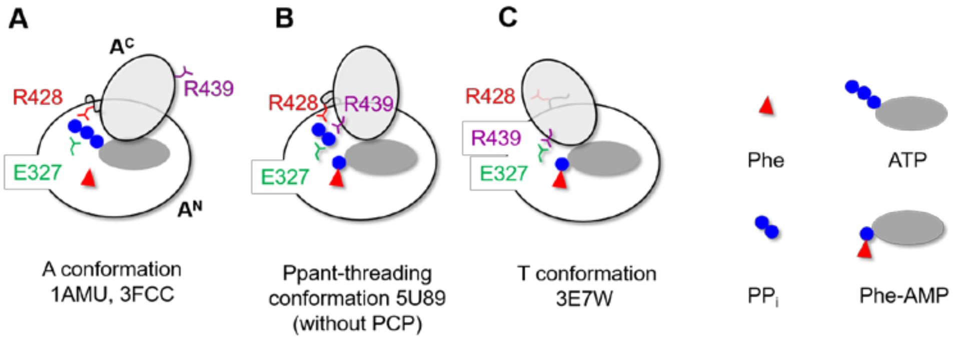

Supplementary Fig. 10 Schematic representations of the A^N and A^C subdomains in the A conformation (A, pdb 1AMU[4], 3FCC)[8], the Ppant-threading I conformation (B, 5U89)[7] and the T conformation (C, 3E7W)[9]. The proposed PP_i binding site of the Ppant-threading I conformation (**B**) is enabled by the opening of the E327-R428 clamp compared to the T conformation (**C**). The residues corresponding to E327, R428 and R439 in the 1AMU structure of GrsA are E298, R397 and R408 for the 3FCC structure, E751, R852 and R862 for the 5U89 structure, as well as E297, R396 and R407 for the 3E7W structure. Note that coordination of the phosphate groups through E327 (E298 in pdb 3FCC) is believed to occur through water-coordinated Mg^{2+} or Ca^{2+} ions, which are not represented in this figure.

Legend for Dataset S1

Dataset S1 (separate file). Overview of data obtained by hydrogen-deuterium exchange mass spectrometry (HDX-MS).

Supplementary References

1. L. Luo, M. D. Burkart, T. Stachelhaus and C. T. Walsh, *J Am Chem Soc*, 2001, **123**, 11208-11218.
2. P. G. Gottschalk and J. R. Dunn, *Anal Biochem*, 2005, **343**, 54-65.
3. M. A. Marahiel, T. Stachelhaus and H. D. Mootz, *Chem Rev*, 1997, **97**, 2651-2674.
4. E. Conti, T. Stachelhaus, M. A. Marahiel and P. Brick, *EMBO J*, 1997, **16**, 4174-4183.
5. C. A. Mitchell, C. Shi, C. C. Aldrich and A. M. Gulick, *Biochemistry*, 2012, **51**, 3252-3263.
6. L. A. Kelley, S. Mezulis, C. M. Yates, M. N. Wass and M. J. Sternberg, *Nat Protoc*, 2015, **10**, 845-858.
7. M. J. Tarry, A. S. Haque, K. H. Bui and T. M. Schmeing, *Structure*, 2017, **25**, 783-793 e784.
8. K. T. Osman, L. Du, Y. He and Y. Luo, *J Mol Biol*, 2009, **388**, 345-355.
9. H. Yonus, P. Neumann, S. Zimmermann, J. J. May, M. A. Marahiel and M. T. Stubbs, *J Biol Chem*, 2008, **283**, 32484-32491.
